# Transcriptomic and fluxomic changes in *Streptomyces lividans* producing heterologous protein

**DOI:** 10.1186/s12934-018-1040-6

**Published:** 2018-12-21

**Authors:** Wouter Daniels, Jeroen Bouvin, Tobias Busche, Christian Rückert, Kenneth Simoens, Spyridoula Karamanou, Lieve Van Mellaert, Ólafur H. Friðjónsson, Bart Nicolai, Anastassios Economou, Jörn Kalinowski, Jozef Anné, Kristel Bernaerts

**Affiliations:** 1 Department of Chemical Engineering, Bio- and Chemical Systems Technology, Reactor Engineering and Safety Section, KU Leuven, Celestijnenlaan 200F, box 2424, 3001 Leuven, Belgium; 2 Center for Biotechnology (CeBiTec), Bielefeld University, Universitätsstraße 27, 33615 Bielefeld, Germany; 3 Department of Microbiology and Immunology, Laboratory of Molecular Bacteriology, KU Leuven, Herestraat 49, box 1037, 3000 Leuven, Belgium; 4 Matís, Vínlandsleid 12, 113 Reykjavík, Iceland; 5 Division of Mechatronics, Biostatistics and Sensors (MeBioS), Department of Biosystems (BIOSYST), KU Leuven, Willem de Croylaan 42, 3001 Leuven, Belgium

**Keywords:** *Streptomyces lividans*, Heterologous protein production and secretion, $^{13}\hbox {C}$-based metabolic flux, RNA-seq analysis, Gene clustering analysis

## Abstract

**Background:**

The Gram-positive *Streptomyces lividans* TK24 is an attractive host for heterologous protein production because of its high capability to secrete proteins—which favors correct folding and facilitates downstream processing—as well as its acceptance of methylated DNA and its low endogeneous protease activity. However, current inconsistencies in protein yields urge for a deeper understanding of the burden of heterologous protein production on the cell. In the current study, transcriptomics and $^{13}\hbox {C}$-based fluxomics were exploited to uncover gene expression and metabolic flux changes associated with heterologous protein production. The *Rhodothermus marinus* thermostable cellulase A (CelA)—previously shown to be successfully overexpressed in *S. lividans*—was taken as an example protein.

**Results:**

RNA-seq and $^{13}\hbox {C}$-based metabolic flux analysis were performed on a CelA-producing and an empty-plasmid strain under the same conditions. Differential gene expression, followed by cluster analysis based on co-expression and co-localization, identified transcriptomic responses related to secretion-induced stress and DNA damage. Furthermore, the OsdR regulon (previously associated with hypoxia, oxidative stress, intercellular signaling, and morphological development) was consistently upregulated in the CelA-producing strain and exhibited co-expression with isoenzymes from the pentose phosphate pathway linked to secondary metabolism. Increased expression of these isoenzymes matches to increased fluxes in the pentose phosphate pathway. Additionally, flux maps of the central carbon metabolism show increased flux through the tricarboxylic acid cycle in the CelA-producing strain. Redirection of fluxes in the CelA-producing strain leads to higher production of NADPH, which can only partly be attributed to increased secretion.

**Conclusions:**

Transcriptomic and fluxomic changes uncover potential new leads for targeted strain improvement strategies which may ease the secretion stress and metabolic burden associated with heterologous protein synthesis and secretion, and may help create a more consistently performing *S. lividans* strain. Yet, links to secondary metabolism and redox balancing should be further investigated to fully understand the *S. lividans* metabolome under heterologous protein production.

**Electronic supplementary material:**

The online version of this article (10.1186/s12934-018-1040-6) contains supplementary material, which is available to authorized users.

## Background

*Streptomyces lividans* is an attractive host for the heterologous production of both mammalian and microbial proteins, when traditional host systems return unsatisfactory results due to incorrect protein folding or lack of protein expression [1, 2]. A main advantage of Gram-positive bacteria such as *S. lividans* is the direct secretion of correctly folded heterologous proteins into the fermentation broth. *S. lividans* TK24—a plasmid-free derivative of *S. lividans* 66 [3]—is preferred over other highly secreting *Streptomyces* species because of its relatively low level of extracellular protease activity, limited restriction-modification system, and available biochemical knowledge due to its high similarity to the *Streptomyces* model organism *S. coelicolor* [1].

Heterologous protein yields obtained in *S. lividans* are often low or inconsistent, driving the research for uncovering production bottlenecks and applying improvement strategies [1, 4]. Screening for alternative promoters and signal peptides [5], codon optimization [6], and optimization of operational conditions [7] have been applied as strategies—with varying success—for improving protein production in *S. lividans*. Additional increases in protein production might be obtained by finding genetic targets based on a thorough understanding of the metabolic burden caused by recombinant protein production. The presence of such metabolic effects that can be exploited was shown by metabolomics studies on a *S. lividans* strain producing murine Tumour Necrosis Factor-α, and that showed profound changes in its metabolic fingerprint [8], as well as in the activation of overflow metabolism [8, 9].

In the current study, changes in gene expression and metabolic fluxes in *S. lividans* TK24 overproducing the thermostable Cellulase A (CelA) from *Rhodothermus marinus* were investigated. The 260 aminoacyl residues of CelA are preceded by a 28-residue amino-terminal signal peptide of *Streptomyces venezuelae* subtilisin inhibitor (*vsi*), which is cleaved off upon secretion via the Sec pathway. Sec dependent secretion is the major secretion route in *Streptomyces* and is most often used for heterologous protein secretion [1, 4]. Overproduction of secretory proteins dependent on the Tat pathway—the second main pathway in *Streptomyces*, that secretes cytoplasmically pre-folded proteins—was found to cause a stringent response, plausibly negatively affecting productivity. No such response was found when the overproduced protein was Sec dependent [10]. However, such a response may also be dependent on the protein context and the levels of synthesis.

$^{13}\hbox {C}$-based metabolic flux analysis is the method of choice for reliable estimation of metabolic fluxes in the central carbon metabolism [11]. The central carbon metabolism provides precursors, energy and reductive power for anabolic reactions such as protein biosynthesis, and transmembrane transport reactions such as protein secretion. Heterologous protein biosynthesis and secretion will therefore have to compete for resources with the endogenous cell processes. The $^{13}\hbox {C}$-fluxomics experiments were performed in a defined minimal medium containing glucose as the sole carbon source. A well-defined carbon source is an absolute requirement to clearly trace the distribution of $^{13}\hbox {C}$ in the intracellular components, and thus allow metabolic flux estimation. Data were collected during exponential growth, when cells were assumed to be in pseudo steady state—i.e., lacking accumulation or depletion of intracellular metabolites and $^{13}\hbox {C}$ [12]. Replicate experiments are performed and jointly fitted to increase confidence in the flux estimates.

In parallel with $^{13}\hbox {C}$-fluxomics, RNA-sequencing was performed to compare the transcriptomes the CelA-synthesizing strain and the empty plasmid-carrying reference strain. Samples for RNA-seq data were harvested under the same conditions as those for $^{13}\hbox {C}$ fluxomics, i.e., in a minimal medium with glucose under exponential cell growth. A transcriptomic analysis, consisting of differential gene expression analysis and expression-based gene clustering, was performed on obtained RNA-seq data. To obtain informative gene clustering results, additional RNA-seq data are generated for the early-, mid-, and late-exponential, and stationary phase.

Our data reveal CelA-producing *S. lividans* has higher flux through the pentose phosphate pathway (PPP) and tricarboxylic acid cycle (TCA), shows gene expression linked to secretion stress and DNA damage, and shows induced transcription of the OsdR regulon—which is associated with hypoxia, oxidative stress, intercellular signaling and morphological development.

## Methods

### Strains, media and preculturing procedure

Three strains were used: *Streptomyces lividans* TK24, wild type (John Innes Centre, Norwich, UK), *S. lividans* TK24 containing the multi-copy plasmid pIJ486, and *S. lividans* TK24 containing pIJ486 with the *celA* gene of *Rhodothermus marinus* cloned behind the strong constitutive promoter and the signal peptide of *Streptomyces venezuelae* subtilisin inhibitor (*vsi*) [2, 13, 14]. All DNA manipulations were done according to [15]. The plasmid was maintained by addition of 10 mg thiostrepton/L (Merck, Darmstadt, Germany) to all growth media. Mycelium stocks were kept at $-\,80\,^\circ \hbox {C}$ in 20% v/v glycerol

Precultures were prepared in phage medium (per L: 10 g glucose, 5 g tryptone, 5 g LabM (Oxoid, Thermo-Fisher, Merelbeke, Belgium), 5 g yeast extract $0.74\,\hbox {g} \, \hbox {CaCl}_2\cdot 2\hbox {H}_2 \hbox {O}, 0.5\,\hbox {g}\, \hbox {MgSO}_4\cdot 7\hbox {H} _2 \hbox {O}, \,\hbox {pH} \; 7.2$) [16]. Experiments for fluxomics and transcriptomics were performed in a minimal medium containing glucose as sole C-source (MMGLC; per L: 55.5 mmol glucose, 1.8 g $\hbox {NaH}_2{\mathrm{PO}}_4, 2.6\,\hbox {g}\,\hbox {K}_2{\mathrm{HPO}}_4, 0.6\,\hbox {g}\,\hbox {MgSO}_4, 3\,\hbox {g}\,(\hbox {NH}_4)_2{\mathrm{SO}}_4, 1\,\hbox {mg}\,\,\hbox {ZnSO}_4\cdot 7\hbox {H}_2{\mathrm{O}}, 1\,\hbox {mg}\,\hbox {FeSO}_4\cdot 7\hbox {H}_2{\mathrm{O}}, 1\,\hbox {mg}\, \hbox {MnCl}_2\cdot 4\hbox {H}_2{\mathrm{O}}$, and 1 mg $\hbox {CaCl}_2$). For $^{13}\hbox {C}$-based fluxomics experiments, a mixture of 56% uniformly labeled glucose (U-GLC) and 44% position 1-labeled glucose (1-GLC) was used (Cambridge Isotope Laboratories, Tewksbury, Massachusetts, United States). This optimal mixture was determined based on the network model and d-optimal design method in [17]. For expression-based gene clustering, additional samples for transcriptomics were harvested from growth in MMGLC supplemented with 5 g/L BD Bacto™Casamino Acids, Technical (MMGLC+CAS). Other chemicals were purchased from Sigma-Aldrich.

Precultures were subcultured twice in baffled shake flasks with 100 mL phage medium incubated at $30\,^\circ \hbox {C}$ and stirred on a magnetic stirrer (600 rpm). Precultures were grown for 72 h and 24 h, respectively. From the first preculture, 25 mL was collected, centrifuged (20 min at 3200×*g*), supernatant was removed, and the pellet was resuspended in phage medium. For inoculation of the bioreactor, 75 mL of the second preculture was centrifuged (20 min at 3200×*g*), supernatant was removed, the pellet was washed twice with fresh reactor medium and resuspended in 9 mL reactor medium, of which 3 mL was used to inoculate the bioreactor medium.

### Bioreactor experiments

Experiments were performed in a DASGIP parallel bioreactor system (Eppendorf, Jülich, Germany) with a working volume of 1 L. Temperature, pH, agitation speed, and air flow were set at $30\,^\circ \hbox {C}$, 6.8, 500 rpm, and 1 vvm, respectively. Dissolved oxygen was not actively controlled, but never dropped below 30%. Antifoam (Y-30 emulsion, Sigma-Aldrich, Overijse, Belgium) was added to avoid foam formation (500 $\upmu$L/L initially, and further supplemented when required). For fluxomics, duplicate reactors were inoculated from the same preculture. One reactor contained medium with labeled glucose and the other reactor contained medium with unlabeled glucose. The parallel experiment with unlabeled glucose was necessary to obtain representative values for the off-gas analysis, as labeled carbon gave rise to erroneous $\hbox {CO}_2$ concentration values in the infrared off-gas analysis. Parallel runs inoculated from the same preculture are here referred to as technical replicates, while repeat experiments started from different precultures are further referred to as biological replicates. Samples for transcriptome analysis were collected under the same conditions as those of the fluxomics experiments, in either the DASGIP parallel bioreactor system, or in a Bioflo 3000 bioreactor (New Brunswick, New Jersey, USA) containing 3 L medium.

### Biomass and extracellular metabolite analysis

At regular time points, samples were collected to determine cell dry weight and extracellular metabolites. Samples (5 or 10 mL) were centrifuged for 20 min at 3200×*g*, supernatant was filtered over $0.2\,\upmu \hbox {m}$ PES (Filtropur, Sarstedt, Etten-Leur, Netherlands) stored for further analysis at $4\,^\circ \hbox {C}$, and the cell pellet was resuspended in 5 mL of ultra-pure water. Cell dry weight concentration was determined by filtration of the cell pellet suspension over a pre-dried, pre-weighted filter (Porafil $0.2\,\upmu \hbox {m}$ cellulose mixed esters membrane filter, Macherey-Nagel, Hoerdt, France) and cell dry weight was quantified after drying overnight at $105\,^\circ \hbox {C}$. Concentrations of glucose, $\alpha$-ketoglutaric acid, acetic acid, lactic acid, and pyruvic acid in the supernatant were measured on an Agilent 1200 HPLC (Diegem, Belgium) system equipped with an Aminex HPX-87H column (Bio-rad, Temse, Belgium). The column was kept at $40\,^\circ \hbox {C}$ and eluted with 5 mM $\hbox {H}_2\hbox {SO}_4$ at a rate of 0.60 mL/min. Organic acids were detected with UV (Agilent DAD, at 210 nm) [18]. Glucose was detected by refractive index changes (Agilent RID, at $35\,^\circ \hbox {C}$) [18].

The total protein concentration in the supernatant was quantified using a Bradford protein assay [19]. A calibration curve was established based on known bovine serum albumin (Sigma-Aldrich) concentrations.

Quantification of CelA in the extracellular medium was performed by SDS-PAGE using 13.5% acrylamide gel concentration in Tris-HCl buffers. Prior to electrophoresis the samples were concentrated up to tenfold using a Centrifugal Vacuum Concentrator (Labconco, Kansas City, USA) overnight at $20\,^\circ \hbox {C}$. Purified CelA was used as calibration standard for analysis. SDS-PAGE was followed by wet Western blot transfer in 20% methanol to a Amersham Protran nitrocellulose membrane (GE Healthcare, Diegem, Belgium) using a modular Omnipage (BiocomDirect, Bridge of Weir, United Kingdom) system for both electrophoresis and blotting. All techniques were performed according to the protocols provided in the Western Blotting Principles and Methods by GE Healthcare, 2011. After transfer, the membranes were blocked and washed, and subjected to CelA-specific antibodies (rabbit, in-house production; [2]). A chemiluminescent reaction was achieved by use of an anti-rabbit IgG goat antibody cross-linked to horse-radish peroxidase (Jackson ImmunoResearch, Ely, United Kingdom) and the SuperSignal West Pico PLUS kit (Thermo Scientific, Blijswijk, Netherlands). Produced light was measured by ImageQuant LAS 4000 (GE Healthcare, Diegem, Belgium) and analysed with ImageJ 1.50 g software (National Institutes of Health, USA). For quantification of CelA in the second $^{13}\hbox {C}$ experiment, a Bio-Rad Bio-Dot was used according to manufacturers instructions. A Protran nitrocellulose membrane was wedged into the manifold and tightened under vacuum. After filtration of the samples, the membranes where treated as stated above for detection and quantification.

### RNA isolation, cDNA synthesis and RNA-seq analysis

Samples for transcriptomics analyses were collected at different stages of growth throughout the experiments. Samples of 1 mL were centrifuged at 21,000×*g* for 30 s, supernatant was removed, the pellet was frozen in liquid nitrogen, and stored at $-\,80\,^\circ \hbox {C}$.

RNA was extracted with the innuPREP RNA Mini kit (Analytik Jena, Germany) according to the manufacturer’s instructions. Residual DNA was removed by digestion with 10 U RNase-free DNase I (Thermo scientific) for 1 h in the presence of RiboLock RNase inhibitor (Thermo scientific). After DNA digestion, the RNA was again purified with the same kit. RNA quality was checked by Trinean Xpose (Gentbrugge, Belgium) and the Agilent RNA 6000 Nano Kit on an Agilent 2100 Bioanalyzer (Agilent Technologies, Böblingen, Germany). Ribosomal RNA molecules were removed from total RNA with the Ribo-Zero rRNA Removal Kit (Illumina, San Diego, USA) and removal of rRNA was checked with the Agilent RNA 6000 Pico Kit on an Agilent 2100 Bioanalyzer. Libraries of cDNA were prepared with the TruSeq Stranded mRNA Library Prep Kit (Illumina, San Diego, USA), and the resulting cDNA was sequenced paired end on an Illumina MiSeq system using 75 bp read length.

Trimmed reads with a minimum of length of 36 bp were mapped to the *S. lividans* TK24 genome sequence [20] with Bowtie 2 using standard settings [21]. For visualization of read alignments, ReadXplorer 2.1.0 was used [22]. Transcripts per million (TPM) values were calculated based on the raw read counts per coding sequence plus one pseudo read [23]. For replicate experiments, mean TPM values were computed.

In total, 12 transcriptomics data sets were collected in MMGLC (same medium as for $^{13}\hbox {C}$-fluxomics), and 9 transcriptomics data sets were obtained in MMGLC+CAS. Transcriptomics data covered also the wild-type strain.

### Isotopic labeling analysis

For measurement of mass isotopomer distributions in intracellular proteinogenic amino acids, an extraction and derivatization protocol for proteinogenic amino acid analysis was established based on [24–26]. A 1 mL bioreactor cell culture sample was centrifuged for 5 min at 14,000×*g*. After removing the supernatant, the pellet was resuspended in 500 $\upmu \hbox {L}$ HCl, and incubated at $105\,^\circ \hbox {C}$ for 24 h. The hydrolysed material was centrifuged to remove cell debris, and 250 $\upmu \hbox {L}$ was transferred to a new Eppendorf tube. Samples were subsequently dried under $\hbox {N}_2$ flow at $65\,^\circ \hbox {C}$. For derivatization, 75 $\upmu \hbox {L}$ pyridine was added to the dried samples, followed by addition of $75\,\mu \hbox {l}$ MTBSTFA+1% TBDMCS (Sigma-Aldrich). The mixture was incubated for 30 min at $60\,^\circ \hbox {C}$ in a heating block and centrifuged for 5 min at 14,000×*g*. The supernatant was transferred to vials and injected on a Gas chromatography–mass spectrometry (GC–MS) system consisting of a 7890A GC, equipped with a HP-5MS column, and a 5975C VLMSD MS with triple-Axis detector (Agilent). Mass isotopomer distributions (MIDs) were obtained by manual integration of the ion chromatograms [27]. Prior to further analysis, the resulting MIDs were corrected for naturally occurring isotopes [28], as well as for the fraction of naturally labeled biomass originating from the inoculum [29]. The software tool IsoCor [30] was used for the correction of naturally occurring isotopes. Mass isotopomer distributions were determined for proteinogenic amino acids and only fragments which were proven acceptable for $^{13}\hbox {C}$-MFA by [27] were included. Used fragments can be found in Additional file 1. The relative precision of the isotopomer measurements, used as weighing factor during flux optimization, was assumed to be 0.4 mol% [27, 31, 32].

### Differential gene expression and hierarchical cluster analysis

Differential expression between *S. lividans* with pIJ486 and *S. lividans* with pIJ486-*vsi-celA* grown in MMGLC was performed using the DESeq2 R package [33]. p-values were adjusted to control multiple testing using the Benjamini–Hochberg approach [34]. A false discovery rate (FDR) of 0.05 was used for identification of differential expression. A hierarchical cluster analysis was subsequently applied to discover general expression trends in the differentially expressed genes. To facilitate cluster analysis, additional transcriptomics datasets were included. Data were obtained for *S. lividans* TK24 wild type (WT), WT containing pIJ486 (empty plasmid reference strain), and WT containing pIJ486-*vsi-celA* (CelA-producing strain) grown in MMGLC and $\hbox {MMGLC}+\hbox {C}\hbox {AS}$. The z-scored TPM values of all datasets (21 in total) were ordered in an agglomerative hierarchical cluster tree, based on the minimum Euclidean distance.

### Local clustering

Genes were clustered based on co-expression and physical location on the genome for a higher-level view on differential expression. The Euclidean distance between the z-scored TPM values of all datasets (see above) was used as a measure for co-expression between genes. The full distribution of distances between all 7496 genes in the genome was calculated, and genes were matched based on their p-value within this distribution. Genes *i* and *j* (as indexed in order of physical appearance on the genome) were matched if their p-value was equal to or smaller than a given cut-off p-value, that was given by:

1 $$\begin{aligned} p_{co,ij} = 1-e^{\frac{\ln (1-p_0)}{|i-j|}} \end{aligned}$$

in which $|i-j|$ is the physical distance in number of known genes (1 for neighboring genes, 2 if there is one separating gene, etc.), and $p_0$ is the base p-value for matching two neighboring genes. Clusters were formed by grouping all genes that match directly or indirectly (i.e., through a shared match). Since the requirement that all genes that match directly belonged to the same cluster could lead to large heterogeneous clusters (especially for high values of $p_0$), the resulting clusters were split according to a minimal cut routine that accounts for both Euclidean distance and physical location. Clusters were split until all gene links within the resulting clusters had a p-value lower than a given tolerance $p_{tol}$. The extensive description of the developed clustering routine is provided in Additional file 2. For the expression analysis in this paper, a $p_0$ of 0.05 and a $p_{tol}$ of 0.15 were used.

### Estimation of specific growth rates and yields

Biomass data and extracellular metabolite data were used to compute specific growth rates and yields for $^{13}\hbox {C}$-MFA. Biomass data were fitted by multi-phase linear regression as described in [9]. The model identifies a lag phase, one or more exponential phases with corresponding specific growth rate $\mu$ ($\hbox {h}^{-1}$), and a stationary phase. Biomass data of technical replicates were jointly fitted (i.e., model parameter estimates must be identical for both data sets), while biomass data from biological replicates were fitted with identical specific growth rates, but a different lag phase duration, start of the stationary phase, and initial biomass concentration. All parameters and their linear statistics were estimated by using *lsqnonlin* in MATLAB R2012b (The MathWorks, Inc., MA, USA). The lack-of-fit test (F-test) was used to confirm the presence of multiple growth phases ($\hbox {p}<0.05$) [35].

Yield estimates were obtained by fitting a straight line on metabolite (or biomass) concentrations *y* (mmol) and glucose *x* concentrations (expressed in 100 mmol/L) from the exponential growth phase. The slope corresponds to the yield (mmol/100 mmol glucose). Since measurement errors are present on both measurements, Deming regression was used to estimate the yields. In Deming regression [36] (a special case of total least squares), the slope ${\hat{\beta }}$ can be found as:

2 $$\begin{aligned} {\hat{\beta }} =  \frac{(s_{yy}-\lambda s_{xx})+\sqrt{(s_{yy}-\lambda s_{xx})^2+4 \lambda s_{xy}^2}}{2s_{xy}} \end{aligned}$$

3 $$\begin{aligned} s_{xx} = \frac{1}{n-1} \sum (x_i-{\bar{x}})^2 \end{aligned}$$

4 $$\begin{aligned} s_{xy} = \frac{1}{n-1} \sum (x_i-{\bar{x}})(y_i-{\bar{y}}) \end{aligned}$$

5 $$\begin{aligned} s_{yy} = \frac{1}{n-1} \sum (y_i-{\bar{y}})^2 \end{aligned}$$

6 $$\begin{aligned} \lambda = \frac{\sigma _\epsilon ^2}{\sigma _\eta ^2} \end{aligned}$$

with ${\bar{x}}$ and ${\bar{y}}$ the average value of the *x* and *y* variables, *n* the total number of data points, and $\sigma _\eta ^2$ and $\sigma _\epsilon ^2$ the variances of the measurement errors in glucose (*x*) and metabolite (*y*) concentrations, respectively. These values were estimated from previous experiments, in which technical replicates were performed for all exometabolome measurements (data not shown). Standard deviation of the regression coefficients were calculated using the jackknife leave-one-out method, which has been shown to perform well in the case of Deming regression [37].

Pseudo steady-state was assumed during exponential growth such that the oxygen uptake rate (OUR) and carbon dioxide production rate (CPR) could be calculated from the mass balances:

7 $$\begin{aligned} {OUR} = \frac{F_g^{in}}{V} \cdot \left( f_{O_2}^{g_{in}} - \frac{f_{N_2}^{g_{in}} \cdot f_{O_2}^{g_{out}}}{1 - f_{CO_2}^{g_{out}} - f_{O_2}^{g_{out}} - f_{H_2O}^{g_{out}} } \right) \end{aligned}$$

8 $$\begin{aligned} {CPR} = \frac{F_g^{in}}{V} \cdot \left( \frac{f_{N_2}^{g_{in}} \cdot f_{CO_2}^{g_{out}}}{1 - f_{CO_2}^{g_{out}} - f_{O_2}^{g_{out}} - f_{H_2O}^{g_{out}} } - f_{CO_2}^{g_{in}} \right) \end{aligned}$$

with $F_g^{in}$ the ingoing molar air flow rate (calculated from the volumetric flow rate and the ideal gas law), and $f^{g_{in}}$ and $f^{g_{out}}$ the volumetric fraction in the in- and outgoing air, respectively. In all experiments $f_{O_2}^{g_{in}}$, $f_{CO_2}^{g_{in}}$, and $f_{N_2}^{g_{in}}$ were 20.97%, 0.03% and 78.75%, and $f_{H_2O}^{g_{out}}$ was 1.1%. The total $\hbox {O}_2$ consumption and $\hbox {CO}_2$ production were obtained by integration of the OUR and CPR, respectively. By doing so, yield calculations of $\hbox {CO}_2$ and $\hbox {O}_2$ can be executed in analogy with the extracellular metabolites.

### Flux estimation

Flux estimation and linearized statistical analysis were performed in the software package 13CFLUX2 [38]. Monte Carlo simulations were performed in influx_s [39] to determine the non-linear confidence intervals. The network model for the central carbon metabolism was implemented in FTBL-format, which is accepted as input file by both packages (Additional file 3). Details on the model construction are given in Additional file 1. Flux estimates were obtained by minimising the differences between the measured and simulated values of MIDs and net flux measurements via non-linear weighted least squares regression [38]. To ensure a global optimum was reached, flux fittings were started from 100 sets of random, initial flux values. The non-linear confidence intervals were obtained through 200 Monte Carlo runs. Data from replicate experiments were jointly fitted by including all measurements in the FTBL file.

### Calculation of cofactor and energy balances

Cofactor and energy dependencies for the reactions in the $^{13}\hbox {C}$-MFA model were taken from the genome-scale model of *S. lividans* [40]. Glucose-6-phosphate dehydrogenase and isocitrate dehydrogenase are $\hbox {NADP}^+$-dependent, while 6-phosphogluconate, malate, glyceraldehyde 3-phosphate, and pyruvate dehydrogenase are NADH-dependent. Two enzymatic routes convert $\alpha$-ketoglutarate into succinyl-CoA: the NADH-generating 2-oxoglutarate dehydrogenase complex, and the 2-oxoglutarate synthase coupled to ferredoxin-$\hbox {NADP}^+$ reductase, which generates NADPH. Contribution of each reaction pathway was chosen in accordance to their relative expression in the transcriptomics data.

Cofactor (NADPH, NADH) and energy (ATP) production and consumption were calculated based on the estimated fluxes and the metabolic requirements for the synthesis of biomass and protein, respectively (Additional file 4). One gram of biomass required 32.5 mmol and 13.1 mmol of ATP and NADPH, respectively, and resulted in the production of 2.3 mmol NADH. Protein synthesis required 53.8 mmol ATP and 20.9 mmol NADPH per gram, while producing 3.8 mmol NADH. Given pseudo steady-state during exponential growth, production and consumption of cofactors and energy is assumed to be balanced. Excess NADPH was assumed to be re-oxidized via transhydrogenase activity, generating NADH. Excess NADH was re-oxidized through oxidative phosphorylation, generating ATP in accordance to a P/O of 1.5 [40]. Excess ATP was assumed to be used in non-quantifiable cell maintenance processes (mATP), such as turn-over of structural molecules and homeostasis.

## Results

### Differential gene expression and cluster analysis

Differential expression analysis was performed on RNA-seq data of *S. lividans* TK24 carrying either pIJ486 (reference strain) or pIJ486-*vsi-celA* (CelA-producing strain) in the exponential growth phase in a minimal medium with glucose. A set of 173 genes out of a total of 7496 showed differential expression between the reference and producing strain with a Benjamini–Hochberg adjusted p-value smaller than 0.05. Out of these 173 significantly differentially expressed genes, 136 were upregulated and 37 were downregulated in the CelA-producing strain. For this set of differentially expressed genes, a hierarchical cluster analysis was performed using an extended data collection (21 RNA-seq data sets) including data for the wild type, multiple culture phases, and a rich medium. The resulting hierarchical cluster tree was split to form 26 clusters. Unprocessed TPM values and all differentially expressed genes are given in Additional file 5.

**Fig. 1:**
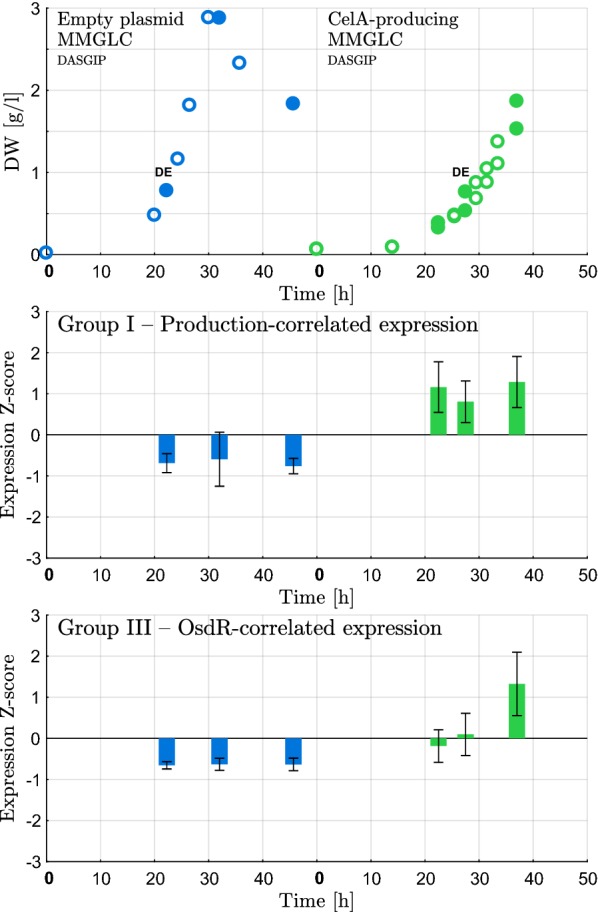
Expression of clusters derived from differential expression analysis. Z-scored expression profiles in the main hierarchical clusters of genes differentially expressed between *S. lividans* TK24 containing pIJ486 and pIJ486-*vsi-celA*, respectively. Filled circles indicate biomass samples for which RNA-seq was performed. DE indicates the data points used for differential expression analysis. Hierarchical cluster expression profiles for all transcriptomics samples used in hierarchical clustering are given in Additional file 5

Three large expression groups were identified from hierarchical clustering: (Group I) 67 genes were significantly activated in a CelA-producing strain, with little expression in data from the wild-type and empty-plasmid strain, (Group II) 42 genes were clustered in a relatively heterogeneous group containing 11 upregulated and 31 downregulated genes, and (Group III) 35 genes had a distinct expression pattern that coincided with the expression of response regulator OsdR. Expression groups I and III were sharply defined and consistent. Group II, in contrast, contained no clear expression trend, and included both upregulated and downregulated genes. Genes in this group were consequently unlikely to be co-expressed as part of a single cellular response. These genes, and the 29 genes not clustered in one of the three large groups (always contained in groups of 5 or less genes) are discussed on an individual level, rather than as being part of a coordinated response. Normalized expression patterns for Group I (CelA production-correlated genes) and III (OsdR-correlated expression) for the producing and reference strain are given in Fig. 1. Expression patterns for all transcriptomics data used for clustering—which includes data from the wild-type strain and data in a medium supplemented with casamino acids—are given in Additional file 5. In addition, genes were grouped using the developed location-based clustering algorithm (Additional file 2). The resulting local gene clusters allowed for a more insightful analysis and functional classification, as co-expression along with shared genomic location is a strong indicator of a functional relation. Results are given in Tables 1, 2, 3, and 4.

**Table 1:** Genes with (partially) known function that are significantly overexpressed in CelA-producing *S. lividans* TK24 with a Benjamini–Hochberg FDR 0.05, and that are only expressed in a CelA-producing strain

Locus		Function	Expression [TPM]			p-value
SLIV	SCO		Ref.	CelA	log2(FC)	
01700	7522	DNA ligase 2	6	36	2.50	7.9e−06
01705	7521	Beta-lactamase	2	12	2.88	3.1e−02
03875	7076	Two-component histidine kinase	5	30	2.72	4.5e−05
03880	7075	Response regulator	21	105	2.29	3.7e−05
04015	7047	Undecaprenyl-diphosphatase 1	7	53	2.87	7.6e−05
06835	6262	Helicase	9	36	1.95	1.5e−04
09765	5770	Regulatory protein RecX	36	172	2.24	5.3e−07
09770	5769	Recombinase RecA	314	1327	2.08	1.9e−11
09810	5761	ATP-dependent DNA helicase	6	19	1.58	7.4e−04
10645	5566	ATP-dependent DNAhelicase RecG	27	63	1.25	1.8e−03
10985	5504	Integral membrane protein	83	476	2.52	8.3e−09
11035	5494	DNA ligase 1	57	103	0.86	1.1e−02*
13140	5049	Putative NADHdehydrogenase/NAD(P)H nitroreductase	0	225	8.84	2.9e−24
13190	5039	Penicillin-binding protein	121	240	0.99	1.1e−03
13680	4938	ECF-sigma factor	1	20	4.99	2.5e−04
13690	4936	ABC transporter ATP-binding protein	3	244	6.16	2.5e−23
13695	4935	Integral membrane protein	2	238	7.25	4.1e−40
17590	4157	Protease HtrB	4	875	7.70	4.0e−63
17595	4156	Response regulator CssR	6	494	6.40	2.5e−37
17600	4155	Sensor histidine kinase CssS	2	101	5.98	1.0e−23
18305	4021	Two component system histidine kinase	1	81	5.82	3.6e−22
18310	4020	Putative transcriptional regulatory protein	16	493	4.95	3.5e−32
18435	3977	Protease HtrA3	62	204	1.71	5.8e−07
19310	3798	Chromosome condensation protein	9	260	4.82	1.5e−24
20575	3542	Integral membrane protein with kinase activity	45	61	0.43	2.7e−01*
20580	3541	DNA polymerase III subunit delta	59	88	0.57	1.1e−01*
20605	3434	DNA polymerase I	11	43	1.97	3.0e−04
21020	3351	DNA repair protein RadA	148	277	0.91	2.6e−03
26430	2258	ABC transporter	22	91	2.07	4.4e−05
26435	2257	Putative Daunorubicin/doxorubicin resistance ATP-binding protein	19	98	2.35	9.4e−06
27700	2003	DNA polymerase I	158	287	0.87	1.8e−03
27885	1966	UvrABC system protein B	59	190	1.69	2.1e−06
27925	1958	UvrABC system protein A	53	123	1.20	2.0e−04
28335	1876	RNA polymerase sigma factor	14	69	2.28	8.2e−03*
28340	1875	Penicillin binding protein	72	456	2.66	2.5e−14
28380	1867	Ectoine hydroxylase	100	778	2.95	7.8e−20
28385	1866	l-Ectoine synthase	221	2079	3.24	5.3e−21
28390	1865	Diaminobutyrate-2-oxoglutarate transaminase	24	169	2.81	1.9e−11
28395	1864	l-2,4-Diaminobutyric acid acetyltransferase	30	118	2.00	5.5e−04
31060	1343	Uracil-DNA glycosylase 2 Ung2	191	394	1.04	1.7e−03

A clustering algorithm was used for grouping nearby genes with similar expression patterns. For all *S. lividans* genes, the corresponding ortholog of the model organism *S. coelicolor* is given

* Genes included for completeness, but p-value does not satisfy FDR 0.05

Many of the 67 genes that clustered with the presence and production of CelA (Group I; Fig. 1) are linked to DNA damage repair, protein secretory stress, and antibiotic resistance. Table 1 shows an overview of the CelA-correlated genes with (partially) known function. Among the gene functions linked to DNA damage repair were recombinase RecA and its regulator RecX, repair protein RadA, nucleotide excision endonuclease system UvrABC proteins A and B, Uracil-DNA glycosylase Ung2, two ligases, two polymerases, two helicases, and a chromosome condensation protein. Secretion stress caused the activation of the CssRS two-component system, which was previously shown to regulate extracellular surface-bound proteases HtrB, HtrA1 and HtrA2 in *S. lividans* and found to be induced by secretory protein oversynthesis, more specifically by the presence of incorrectly folded protein outside the cytoplasm [41]. Significant overexpression of *cssRS* neighbor *htrB* was indeed observed in the CelA-producing strain, but induction of both *htrA1* and *htrA2* was not found. The nearby SLIV_18305+18310 two-component system of unknown function was also activated in the CelA-producing strain. An additional protease, HtrA3, was shown to be unaffected by CssRS in *S. coelicolor* by [41], but was found to be significantly overexpressed in our CelA-producing strain. Two penicillin-binding proteins were activated, as well as the putative antibiotics efflux pump SLIV_26430+26435. Furthermore, four genes for ectoine biosynthesis had significantly increased expression.

**Table 2:** *S. lividans* TK24 genes with (partially) known function with expression correlating to that of the OsdR regulatory protein, and significantly higher expression in a CelA-producing strain in exponential phase (Benjamini–Hochberg FDR 0.05)

Locus		Function	Expression [TPM]			p-value
SLIV	SCO		Ref.	CelA	log2(FC)	
01755	7511	Glyceraldehyde-3-phosphate dehydrogenase Gap3	52	323	2.65	2.8e−08
05195	6663	Transketolase	25	207	3.06	4.0e−13
05200	6662	Transaldolase 1	53	158	1.57	3.6e−04
05205	6661	Glucose-6-phosphate 1-dehydrogenase	40	110	1.46	1.9e−04
05215	6659	Glucose-6-phosphate isomerase 1	64	134	1.06	2.4e−03
05220	6658	6-phosphogluconate dehydrogenase	144	221	0.62	4.0e−02*
05225	6657	Membrane-associated oxidoreductase	26	33	0.32	3.9e−01*
18590	3946	CydB cytochrome d ubiquinol oxidase, subunit II	127	204	0.68	2.4e−02*
18595	3945	CydA cytochrome oxidase subunit I	250	892	1.83	2.0e−09
33310	0924	Cytochrome B subunit	45	273	2.59	5.9e−09
33315	0923	Fumarate dehydrogenase	17	96	2.50	3.9e−09
33320	0922	Fumarate dehydrogenase	26	78	1.59	3.1e−03
36805	0219	Respiratory nitrate reductase, gamma subunit	0	28	Inf	5.2e−07
36810	0218	Nitrate reductase subunit delta	0	36	Inf	2.0e−08
36815	0217	Nitrate reductase beta chain	0	111	8.23	1.0e−28
36820	0216	Nitrate reductase alpha subunit	2	192	6.86	3.5e−49
36825	0215	$\hbox {Nitroreductase}^{\dagger }$	1	82	7.14	4.0e−15
36830	0214	Pyridoxamine 5′-phosphate $\hbox {oxidase}^{\dagger }$	4	58	4.03	1.4e−04
36835	0213	Nitrate/nitrite transporter	3	114	5.46	1.0e−20
36845	0211	Two-component sensor	2	73	5.21	1.4e−08
36850	0209	Cystathionine $\beta$-$\hbox {syntase}^{\dagger }$	1	109	6.97	1.4e−20
36855	0208	Pyruvate, phosphate dikinase	1	65	5.90	5.4e−24
36865	0206	Putative pyruvate formate-lyase	0	74	Inf	3.1e−05
36867		Putative pyruvate formate-lyase-activating protein, N-terminal fragment, putative pseudogene	0	74	Inf	7.7e−06
36875	0204	Transcriptional regulatory protein OsdR	16	391	4.60	3.1e−25
36880	0203	Two-component sensor OsdK	23	56	1.30	4.2e−03*
36890	0201	Thiosulfate dehydrogenase (quinone)$^{\dagger }$	1	464	8.97	3.5e−37
36895	0200	Universal stress $\hbox {protein}^{\dagger }$	0	317	Inf	2.5e−04
36900	0199	Alcohol dehydrogenase	1	214	8.55	9.3e−26
36905	0198	Universal stress $\hbox {protein}^{\dagger }$	1	72	6.16	2.9e−11
36910	0197	Pyridoxamine 5′-phosphate $\hbox {oxidase}^{\dagger }$	6	97	4.07	3.1e−09
37035	0181	Universal stress $\hbox {protein}^{\dagger }$	2	175	6.76	4.0e−22
37040	0180	Universal stress $\hbox {protein}^{\dagger }$	0	69	Inf	2.7e−14
37045	0179	Zinc-containing dehydrogenase	4	293	6.37	6.9e−32
37050	0178	Universal stress $\hbox {protein}^{\dagger }$	8	28	1.72	1.2e−01
37060	0177	Membrane protein	2	112	6.22	2.5e−12
37070	0174	pyridoxamine 5′-phosphate $\hbox {oxidase}^{\dagger }$	1	232	8.50	6.0e−32
37075	0173	OsmC regulator of disulfide bond formation redox $\hbox {protein}^{\dagger }$	7	78	3.48	2.8e−06
37080	0172	Universal stress $\hbox {protein}^{\dagger }$	3	17	2.76	1.1e−01
37085	0171	Nicotinate phosphoribosyltransferase	0	56	Inf	3.5e−17
37095	0169	Cystathionine $\beta$-$\hbox {synthase}^{\dagger }$	10	398	5.29	4.0e−30
37100	0168	Regulator protein	10	625	5.94	5.1e−33
37105	0167	Universal stress $\hbox {protein}^{\dagger }$	4	297	6.35	8.5e−31
37130	0162	$\hbox {Nitroreductase}^{\dagger }$	0	91	8.28	3.5e−17
37090	0170	Cystathionine $\beta$-$\hbox {synthase}^{\dagger }$	6	252	5.44	1.2e−21

A clustering algorithm was used for grouping nearby genes with similar expression patterns. When present, the corresponding ortholog of the model organism *S. coelicolor* is given

* Genes included for completeness, but p-value does not satisfy FDR 0.05

^†^Function taken from [42]

In Group III (Fig. 1), a total of 35 differentially expressed genes followed a distinct expression pattern over time: low base expression, increasing expression over the exponential phase in a CelA-producing strain in a minimal medium, and strong but transient expression in the late exponential phase in a medium containing casamino-acids (Additional file 5). The Group III genes are given in Table 2. The group of genes on the right arm of the chromosome (SLIV_36805 to SLIV_37130) represents the OsdR regulon (oxygen availability, stress, and development; also called DevR, in analogy to the *Mycobacterium tuberculosis* dormancy regulator) [42]. The OsdR regulon in *Streptomyces* is not fully understood, but in the model organism *S. coelicolor* it has been linked to hypoxia [43, 44], nitrate respiration [44], response to oxidative and other forms of stress [42], and intercellular signaling through control of cellular homeostasis of nitric oxide, nitrite and nitrate—regulating morphological differentiation by delaying development of aerial mycelia [42, 43] and sporulation [42, 44], and by inducing production of the antibiotic undecylprodigiosin [43]. CydA and CydB are the subunits of an alternative terminal oxidase—a *bd*-type menaquinol oxidase—that has a higher affinity for oxygen, enabling oxidative phosphorylation at limited oxygen concentrations at the cost of lower respiratory efficiency. Cytochrome *bd* has also been shown to protect from oxidative and nitrosative stress [45, 46]. The SCO0922:0924 (SLIV_33310:33320) operon has previously been reported to be co-expressed with CydAB, and was suggested to encode fumarate reductase, which shows to be active together with cytochrome *bd* under microaerobic conditions in *S. coelicolor* and *M. tuberculosis* [47, 48]. The SLIV_05195:05225 genes in turn correspond to the secondary isoenzymes for the PPP, suggested to provide energy for secondary metabolism [49]. Also involved in the central carbon metabolism is SLIV_01755, which codes for a secondary glyceraldehyde 3-phosphate dehydrogenase (GAPDH) Gap3 [50, 51].

**Table 3:** *S. lividans* TK24 genes with (partially) known function with significantly higher expression in a CelA-producing strain compared to an empty plasmid-containing strain in early exponential phase (Benjamini–Hochberg FDR 0.05), but of which expression can not solely be contributed to CelA production

Locus		Function	Expression [TPM]			p-value
SLIV	SCO		Ref.	CelA	log2(FC)	
07685	6091	Integral membrane protein	30	70	1.24	2.4e−03
22330	3073	Urocanate hydratase	4	37	3.14	5.3e−07
12430	5190	DNA-binding protein	35	165	2.24	1.5e−04
29930	1559	Methionine import ATP-binding protein MetN	26	75	1.52	2.2e−03
29940	1557	Lipoprotein	130	384	1.56	6.0e−05
01630	7536	Integral membrane protein	34	164	2.28	1.4e−09
09290	5863	Sensor protein CutS	11	37	1.82	2.7e−03
11255	5450	ABC transporter	2	18	3.15	7.7e−05
12070	5259	Permease	12	54	2.17	6.3e−04
12810	5114	ABC transporter integral membrane protein BldKC	43	125	1.52	4.5e−04
12815	5113	ABC transporter lipoprotein BldKB	168	314	0.90	1.7e−03
13790	4917	Purine nucleoside phosphorylase	5	64	3.70	3.6e−06
13805	4914	Deoxyribose-phosphate aldolase	7	69	3.24	1.1e−06
13870	4901	Adenosine deaminase 1	11	49	2.16	3.9e−04
16725	4334	Integral membrane protein	8	29	1.95	3.5e−03
16965	4286	Solute-binding protein	12	43	1.90	1.6e−03
17390	4198	DNA-binding protein	170	508	1.58	1.1e−04
18135	4054	Integral membrane protein	24	75	1.65	2.9e−03
19300	3800	Acyl-CoA dehydrogenase	9	56	2.67	2.6e−07
21345	3289	Large membrane protein	12	49	2.04	3.0e−03
22130	3111	ABC transport system ATP-binding protein	37	122	1.74	2.9e−04
22135	3110	ABC transport system integral membrane protein	21	71	1.78	7.6e−06
22235	3090	ABC transporter	55	149	1.45	1.6e−05
22240	3089	ABC transporter ATP-binding protein	87	246	1.51	9.3e−05
23095	2920	Secreted protease	38	116	1.60	3.0e−05
23520	2829	Amino acid ABC transporter transmembrane protein	9	48	2.47	1.3e−03
26890	2164	Integral membrane efflux protein	31	65	1.05	3.4e−03
28875	1773	Alanine dehydrogenase	4	78	4.15	2.3e−11
29635	1621	Glycine/betaine transport ATP-binding protein	6	46	2.96	1.5e−05
29640	1620	Glycine/betaine transport system permease	3	40	3.85	4.4e−12

A clustering algorithm was used for grouping nearby genes with similar expression patterns. When present, the corresponding ortholog of the model organism *S. coelicolor* is given

**Table 4:** *S. lividans* TK24 genes with (partially) known function with significantly lower expression in a CelA-producing strain compared to an empty plasmid-containing strain in early exponential phase (Benjamini–Hochberg FDR 0.05)

Locus		Function	Expression [TPM]			p-value
SLIV	SCO		Ref.	CelA	log2(FC)	
01030	7657	Secreted protein	1297	430	$-$ 1.59	2.2e−04
01080	7647	Calcium-binding protein	251	54	$-$ 2.22	1.7e−03
01125	7638	Enolase 2	197	40	$-$ 2.30	4.4e−05
09410	5839	Peptidase	486	140	$-$ 1.80	4.0e−04
09470	5827	Transmembrane transporter	227	47	$-$ 2.28	3.7e−06
09475	5826	Putative membrane protein	396	124	$-$ 1.67	3.0e−03
10950	5511	Membrane associated phosphodiesterase	232	75	$-$ 1.62	4.1e−04
12120	5249	Nucleotide-binding protein	944	305	$-$ 1.63	5.2e−04
12275	5218	Integral membrane protein	182	43	$-$ 2.08	3.8e−04
12510	5174	Transferase	217	71	$-$ 1.61	1.7e−03
14370	4798	Peptidase	125	37	$-$ 1.75	2.3e−03
17195	4239	Small membrane protein	531	146	$-$ 1.86	1.1e−03
17200	4238	Guanyltransferase	284	81	$-$ 1.82	1.9e−03
18400	4002	Structural cell wall protein NepA	5745	1211	$-$ 2.25	2.4e−07
18605	3943	Transcriptional regulator	285	88	$-$ 1.70	1.9e−03
18635	3926	Sporulation control protein SsgA	175	30	$-$ 2.55	2.1e−03
20395	3579	WhiB-family transcriptional regulator	1517	449	$-$ 1.76	1.2e−04
20705	3413	HTH-type transcriptional activator TipA	435	14	$-$ 4.97	5.3e−18
21025	3350	Alanine-rich protein	57	11	$-$ 2.37	1.5e−03
21180	3323	ECF sigma factor BldN	1502	445	$-$ 1.76	3.9e−05
23265	2884	Cytochrome P450	190	51	$-$ 1.89	6.6e−04
24080	2718	Hydrophobic surface protein	170	20	$-$ 3.06	6.7e−04
28720	1800	Hydrophobic surface protein	10,102	3979	$-$ 1.34	1.3e−03
29370	1674	Hydrophobic surface protein	24,089	8481	$-$ 1.51	4.1e−04
31930	1174	Putative aldehyde dehydrogenase	245	43	$-$ 2.53	4.8e−07
32840	6393	Transposase	299	57	$-$ 2.40	9.1e−07
34120	0762	Protease inhibitor protein	1495	485	$-$ 1.62	1.1e−03
35005	0588	Sensor kinase	113	34	$-$ 1.74	3.4e−03

A clustering algorithm was used for grouping nearby genes with similar expression patterns. When present, the corresponding ortholog of the model organism *S. coelicolor* is given

**Fig. 2:**
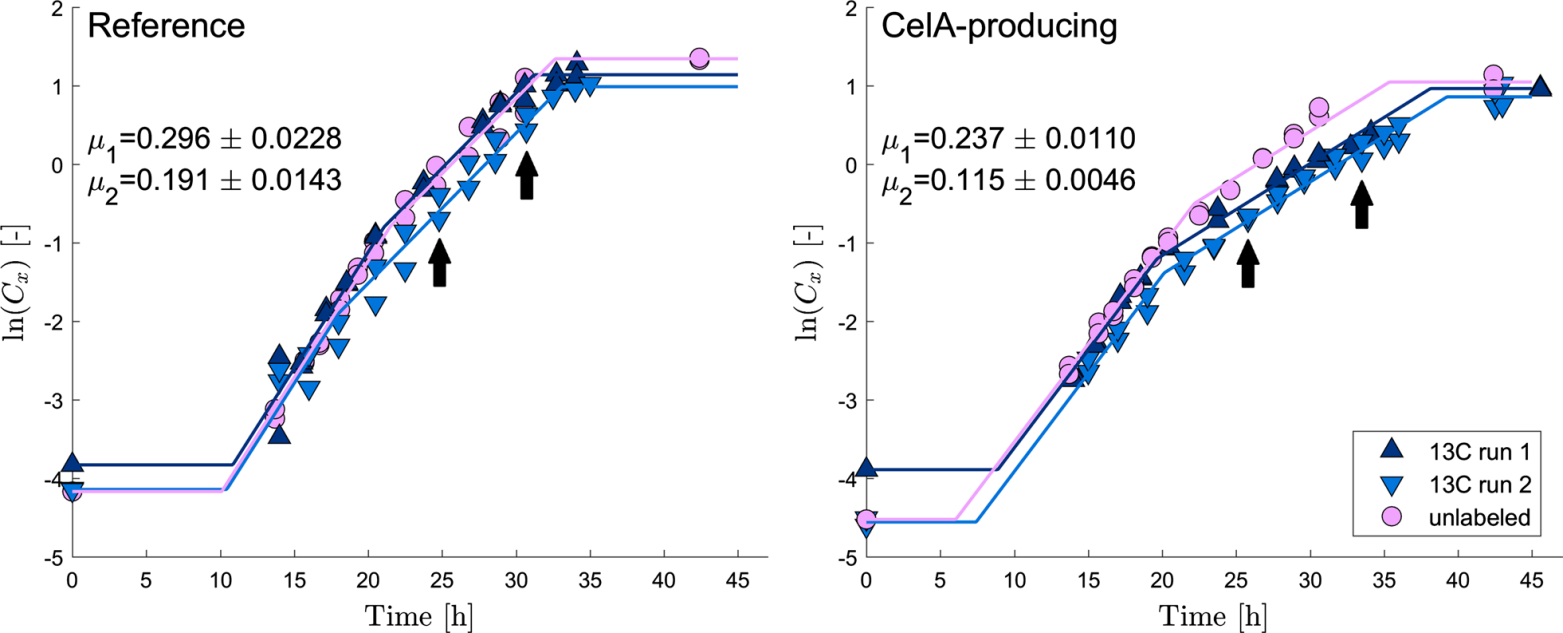
Biomass data of repeated batch experiments of *S. lividans* TK24 containing pIJ486 and pIJ486-*vsi–celA* used for $^{13}\hbox {C}$- fluxomics. The average standard deviation on the biomass concentration measurements ($\hbox {gDW}\,\hbox {L}^{-1}$) is 0.20. Specific growth rates are given along with their standard deviations. Time points analyzed for isotopic labeling in intracellular proteinogenic amino acids are indicated. Flux maps are computed for the second time point. A multi-linear regression according to [9] is performed to identify exponential growth phases

Additional genes that were overexpressed in the producing strain (exponential phase, MMGLC), but where expression was not limited to the CelA-producing strain (Group I) or correlated to *osdR* expression (Group III), are given in Table 3, while all genes that were underexpressed under these conditions are given in Table 4. Underexpressed genes notably included the sporulation control protein SsgA, and developmental sigma factor gene *bldN* and five of its known targets: three hydrophobic cell-surface proteins known as chaplins (SLIV_24080, SLIV_28720 and SLIV_29370), structural cell wall protein NepA, and a transferase (SLIV_12510) [52, 53]. Expression of *bldN* has been linked to OsdR and its role in delaying aerial mycelia development, though the exact mechanism remains elusive [42].

Overall, differential expression and hierarchical clustering revealed two large expression trends in CelA-producing *S. lividans*: a stress response mainly linked to secretory stress and DNA damage, and increasing expression throughout the exponential phase in MMGLC of a co-expressed group of genes containing response regulator OsdR. The latter included both the known OsdR regulon, as well as a set of enzymes of which most are linked to central carbon metabolism.

**Table 5:** Estimated yields per 100 mmol glucose for the CelA-producing and the empty-plasmid *S. lividans* based on concentration measurements from repeated experiments in minimal medium with glucose (MMGLC)

Yield (per 100 mmol glucose)*	*S. lividans* with *pIJ486*		*S. lividans* with *pIJ486-vsi-celA*	
	Experiment 1	Experiment 2	Experiment 1	Experiment 2
DW (g)*	7.67 ($\pm \,0.26$)	9.31 ($\pm \,0.19$)	4.96 ($\pm \,0.17$)	6.99 ($\pm \,0.21$)
$\hbox {CO}_2$ (mmol)*	213.38 ($\pm \,7.47$)	213.55 ($\pm \,88.31$)	246.07 ($\pm \,13.14$)	182.61 ($\pm \,9.67$)
ACE (mmol)*	8.71 ($\pm \,3.33$)	5.97 ($\pm \,4.07$)	22.64 ($\pm \,0.60$)	28.48 ($\pm \,3.73$)
AKG (mmol)*	0.11 ($\pm \,0.01$)	0.44 ($\pm \,0.27$)	0.24 ($\pm \,0.04$)	0.47 ($\pm \,0.04$)
LAC (mmol)*	1.36 ($\pm \,0.24$)	1.25 ($\pm \,0.27$)	1.37 ($\pm \,0.12$)	1.62 ($\pm \,0.09$)
PYR (mmol)*	0.11 ($\pm \,0.03$)	0.86 ($\pm \,0.57$)	8.56 ($\pm \,0.28$)	16.96 ($\pm \,2.24$)
SUCC (mmol)*	0.10 ($\pm \,0.01$)	0.00 ($\pm \,0.00$)	0.18 ($\pm \,0.03$)	0.15 ($\pm \,0.01$)
Protein (mg)*	59.59 ($\pm \,8.16$)	94.30 ($\pm \,4.34$)	118.43 ($\pm \,12.56$)	119.89 ($\pm \,6.38$)
CelA (mg)*	nd	nd	20.47 ($\pm \,5.66$)	16.96 ($\pm \,2.25$)
C recovery	0.89 ($\pm \,0.02$)	0.99 ($\pm \,0.15$)	0.86 ($\pm \,0.02$)	0.95 ($\pm \,0.03$)

Standard deviations are given between brackets and the carbon recovery per experiment is calculated. The carbon content of the biomass was determined through elemental analysis, and found to be 35.17 ($\pm 0.45$) mmol/gDW

Dry weight (DW), carbon dioxide ($\hbox {CO}_2$), acetic acid (ACE), $\alpha$-ketoglutaric acid (AKG), lactic acid (LAC), pyruvic acid (PYR), succinic acid (SUCC), total secreted proteins (Protein), and secreted CelA, *nd* not determined

**Fig. 3:**
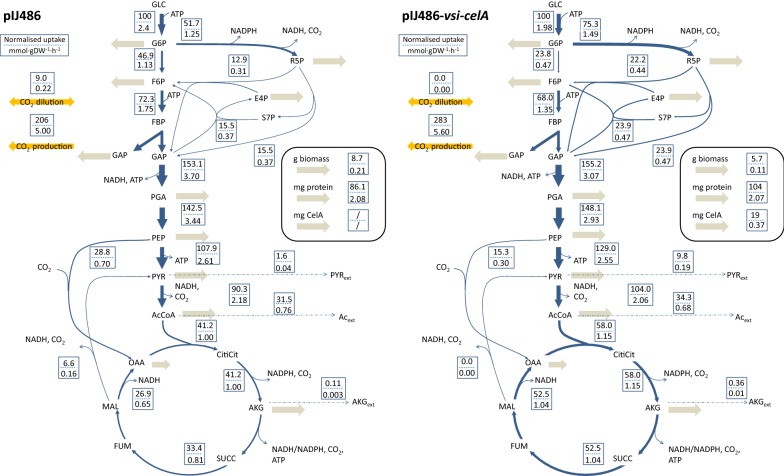
$^{13}\hbox {C}$-based flux maps for *S. lividans* containing pIJ486 (left) and pIJ486-*vsi-celA* (right). Normalized flux estimates and absolute values are given. Estimated fluxes are expressed in mmol per 100 mmol glucose uptake. Yields of biomass or secreted protein are expressed in grams or milligrams per 100 mmol glucose, respectively

**Table 6:** Calculated NADPH, NADH and ATP production and consumption (mmol/g biomass) based on $^{13}\hbox {C}$-MFA fluxes and biomass and protein synthesis requirements

	*S. lividans* with pIJ486			*S. lividans* with pIJ486-vsi-celA		
	Production	Consumption	${{\Delta }}$	Production	Consumption	${{\Delta }}$
NADPH	11.6	$-$ 13.3	$-$1.7	25.2	$-$ 13.6	11.7
NADH	43.1	0.0	43.1	77.3	0.0	77.3
ATP	33.9	$-$ 52.3	$-$ 18.3	58.9	$-$ 61.7	$-$2.9
mATP	43.8			130.6		
NGAM	8.4			15.0		

A balance is obtained by diverting differences ($\Delta$) to maintenance ATP (mATP), returning the non-growth associated ATP maintenance (NGAM) (mmol gDW^−1^ h^−1^)

**Fig. 4:**
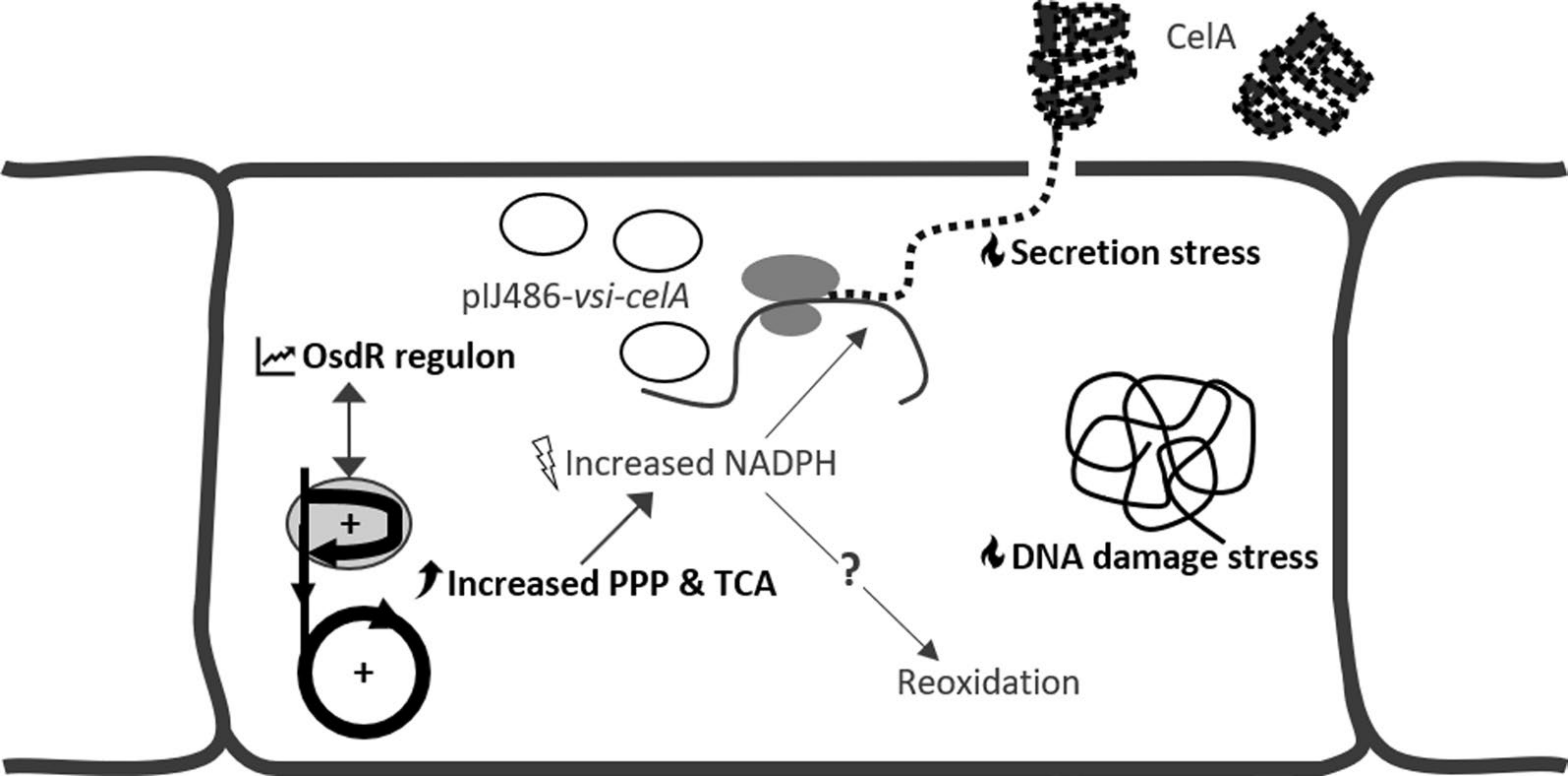
Schematic overview transcriptomic and metabolomic responses in *S. lividans* producing CelA. *S. lividans* containing pIJ486-*vsi-celA* shows stress responses linked to secretion and DNA damage, increased fluxes through the pentose phosphate pathway (PPP) and the tricarboxylic acid (TCA) cycle, and increasing expression of the OsdR regulon throughout the exponential phase. A series of PPP enzymes linked to secondary metabolism was found to be co-expressed with the OsdR regulon. The increased fluxes through the PPP and TCA cycle lead to an increased NADPH production, which can only be partly be attributed to higher NADPH need for additional protein synthesis. Surplus NADPH is assumed to be reoxidized, yet the mechanism for reoxidation of NADPH in *S. lividans* remains unknown

### $^{13}\hbox {C}$-based metabolic flux analysis

Biomass profiles of *S. lividans* respectively carrying pIJ486 and pIJ486-*vsi-CelA* are shown in Fig. 2. Multiple batch experiments with both strains grown on a minimal medium with glucose were performed and two biological repetitions were cultured on an optimal mixture of $^{13}\hbox {C}$-labeled glucose (56% U-GLC and 44% 1-GLC). Repeated experiments showed consistent specific growth rates. Two growth phases were identified by multi-linear regression, where the second phase coincided with the most pronounced metabolite changes (see Additional file 6). Although not confirmed, preculture effects presumably played a role in the first growth phase. Since these effects obscure $^{13}\hbox {C}$-based flux analysis, this phase was not further considered. The specific growth in the second exponential phase was 0.191 $\hbox {h}^{-1}$ for the reference strain, and $0.115~\hbox {h}^{-1}$ for the CelA-producing strain, which was significantly lower (Fig. 2).

Yields for biomass, metabolites, cellulase A, and secreted proteins were computed for the second exponential growth phase for both experiments and are given in Table 5. Carbon recoveries are also given in Table 5, and were acceptable for the first round of experiments and very good for the second round of experiments. The original concentration profiles are available in Additional file 6. Cellulase A production was observed, with the final concentration reaching 7.5 mg/L.

Besides CelA secretion, the total protein secretion increased in the CelA-producing strain, with CelA only making up approximately 16% of the total amount of secreted proteins. Furthermore, a lower biomass yield (33%; Table 5) and lower glucose uptake rate (20%) were observed for the CelA-producing strain compared to the empty-plasmid strain.

Organic acids were secreted by both strains. Acetic, $\alpha$-ketoglutaric, lactic and pyruvic acid were clearly detected, with pyruvic acid representing the largest difference between both strains. Production of succinic acid was negligible. Secretion of acetic acid and pyruvic acid was much higher in the CelA-producing strain, demonstrating a metabolic flux shift.

Mass isotopomer distributions (MIDs) of intracellular protein-bound amino acids were determined in the exponential growth phase. Flux maps were computed for one time point in the second exponential growth phase, indicated in Fig. 2. Isotopic steady state could be observed in time series MID data (data not shown). To avoid biases in the MIDs due to the different metabolic state in the first growth phase, MIDs were corrected with MIDs measured at the start of the second growth phase. The MIDs are given in Additional file 7. Data sets of repeated experiments were jointly fitted to maximize the information content and parameter estimation confidence. Estimated values for the free fluxes and associated non-linear confidence intervals are summarized in Additional file 8. These values were used to compute fluxes for all reactions, which are summarized in flux maps.

Flux maps for the central carbon metabolism in the CelA-producing and empty-plasmid strain are depicted in Fig. 3. Main internal differences between both strains were situated at the level of the glycolysis/PPP split ratio, the TCA fluxes, and the anaplerotic fluxes. Compared to the reference strain, the flux through the oxidative part of the PPP increased from 51.7 to 75.3%, leading to a shift in split ratio between glycolysis and the PPP from 48/52 to 35/75 in the producing strain. The fluxes through the non-oxidative part of the PPP were 50 to 70% higher in the CelA-producing strain. Although the growth rate of the CelA-producing strain was lowered, all fluxes in the TCA cycle increased over 40%. In the CelA-producing strain, phosphoenolpyruvate carboxylase solely replenishes the TCA.

Production and consumption of reductive power (NADPH, NADH) and energy (ATP) were deduced from the carbon flux distributions. A distribution of fluxes between the alternative reactions 2-oxoglutarate dehydrogenase complex ($\hbox {NAD}^+$-dependent) and the 2-oxoglutarate synthase ($\hbox {NADP}^+$-dependent) was deduced from gene expression levels, found to be 78% and 22%, respectively. The P/O ratio was fixed at 1.5 in accordance with [9, 54]. Excess NADH was assumed to be completely re-oxidized via oxidative phosphorylation (thus generating ATP). In the CelA-producing strain, the overproduction of NADPH was assumed to be converted to NADH via transhydrogenase activity. The final results are shown in Table 6. In the reference strain, NADPH production and consumption were nearly balanced. The difference in NADPH could be compensated by NADPH generated via PntAB. The excess NADPH in the CelA-producing strain eventually leads to a higher estimation of the maintenance ATP. The ATP balance was closed by assuming that the total ATP production was split between ATP-requirements for biomass and protein biosynthesis on the one hand, and cell maintenance on the other hand. Consequently, the non-growth associated maintenance ATP-requirements were 15.0 and $8.5 \hbox {mmol}\cdot \hbox {gDW}^{-1}\cdot \hbox {h}^{-1}$ for the producing and reference strains, respectively.

To link metabolic and gene expression changes, all TPM values of genes coding for enzymes catalysing metabolic reactions in the central carbon metabolic network model are given in Additional file 9. Genes of several (iso)enzymes of PPP reactions—e.g., glucose-6-*P*-dehydrogenase, transketolase, and transaldose—were upregulated, which is consistent with the increased metabolic flux through these reactions. Furthermore, both pyruvate dikinase and GAPDH enzyme Gap3 were significantly upregulated and clustered with the OsdR regulon. Pyruvate dikinase overexpression—though limited in absolute value—suggests a flux from pyruvate back to phosphoenolpyruvate, yet the normalized net flux towards pyruvate was found to be higher. Expression of Gap3 ($\hbox {SLIV}\_01755$) was much lower than primary GAPDH enzyme Gap1 ($\hbox {SLIV}\_27980$), and no significant flux change was detected. A third GAPDH enzyme, Gap2 ($\hbox {SLIV}\_04050$), is associated with gluconeogenesis [50] and not expressed in the exponential phase. All of the genes coding for these differentially expressed enzymes clustered with the OsdR regulon, as shown in Table 2. Significantly lower expression was only detected for an enolase isoenzyme, but the normalized flux through this pathway is increased rather than decreased. Finally, although TCA fluxes increased, expression of genes coding for TCA reactions were not significantly changed in the CelA-producing strain.

In conclusion, the CelA-producing strain was found to be slower growing, with increased secretion of organic acid and non-CelA proteins. It showed a higher flux through the oxidative PPP and the TCA cycle, seemingly leading to NADPH overproduction. The increased flux through the PPP corresponded to the significantly increased expression of a group of PPP enzymes. A visual overview of the observed phenomena is given in Fig. 4.

## Discussion

We set out to determine the changes in gene expression and central carbon fluxes resulting from heterologous production and secretion of thermostable cellulase CelA in *S. lividans* through RNA-seq and $^{13}\hbox {C}$-MFA on a both a strain containing pIJ486-*vsi-CelA* and a reference strain containing empty pIJ486. CelA production negatively impacts growth, and results in increased secretion of organic acids (Table 5). The increased organic acid secretion agrees with the study on recombinant *S. lividans* heterologously producing $\hbox {mTNF}\alpha$, where metabolomics revealed that production of the recombinant protein lead to organic acid overflow [8]. However, a significant change in growth between the $\hbox {mTNF}\alpha$-producing strain and a corresponding empty-plasmid reference strain was not observed [8], suggesting the increased organic acid secretion and reduced growth in our study might be unrelated.

It is clear that heterologous production and secretion of CelA induces a distinct stress response in *S. lividans* (Table 1), which seems mainly related to secretory stress and, surprisingly, DNA damage. The cause of this (perceived) DNA damage is unclear, as no direct link with heterologous protein production has been documented in literature. A better understanding of the production-induced stress, and its effect on product yields and cell integrity could lead to identification and mitigation of production bottlenecks.

The presence of secretory stress is not unexpected, and could be directly linked to CelA overproduction and secretion, though it should be noted that the CelA-producing strain also shows an increase in non-CelA protein secretion (Table 5). Secretory stress was previously also observed in *S. lividans* overexpressing its native $\alpha$-amylase AmlB, leading to induction of the CssRS–HtrB protease system [41, 55]. In [41, 55], two additional HtrA-like proteases—HtrA1 and HtrA2—are found to be controlled by CssRS. The Htr protease system is under delicate control, since deletion or overexpression of any of the three proteases, as well as deletion of *cssR* or *cssS* resulted in severe reduction of AmlB enzymatic activity [41, 55]. On top of degradation of incorrectly folded proteins, the system might include chaperone-like activities—promoting correct protein (re)folding [56]. In our study, HtrA1 and HtrA2 have very low and constitutive expression, respectively, and show no sign of being induced by CelA production. A third potential HtrA-like gene, SLIV_18435, which was found to not to be influenced by CssRS in [41], is significantly induced by CelA production in our study (Table 1). Understanding the proofreading system for correct folding of proteins secreted through the Sec pathway—where protein folding occurs upon secretion—could prove essential for obtaining a high-producing *S. lividans*, but requires further study.

Increased expression of four ectoine biosynthesis genes (SLIV_28380:28395; Table 1) would typically imply the presence of osmotic stress. However, a study in *S. coelicolor* shows that three of these gene products—SCO1865:1867—are targeted to the membrane compartment [57], where ectoine may promote stability of membrane proteins [58]. Hence, the expression of these genes in our study may be related to secretion-induced membrane stress rather than osmotic stress.

The increasing expression of the OsdR regulon in the CelA-producing strain cannot easily be explained, and is obfuscated by the plethora of functions attributed to this regulon. The transient high expression in a medium containing casamino acids (Additional file 5) agrees with a cell signalling/regulatory function [42, 43], while the rising expression throughout the exponential phase in the producing strain seems to correspond more to a form of increasing stress. Transcription of *osdR* is induced under both hypoxia and oxidative stress, but a clear link to heterologous protein production is missing. The upregulation of the cytochrome *bd* and fumarate reductase operons—known to be co-expressed in *M. tuberculosis* and *S. coelicolor*—suggests respiration under microaerobic conditions [47, 48]. Whether their co-expression with the hypoxia-linked OsdR regulon [43, 44] entails a direct regulatory interaction, or that an environmental activator is shared in the form of (local) hypoxic conditions, remains unclear. Though a lower growth rate and an increased yield of lactate—a known marker for microaerobic growth conditions [9, 54, 59]—in the CelA-producing strain (Table 5) may be seen as indicators, the evidence for hypoxia can not be truly confirmed. Further research is required to (*i*) confirm the link between production of CelA—or heterologous protein in general—and the Group III genes (Table 2), and (*ii*) show the presence or absence of microaerobic conditions when the OsdR regulon is activated in a producing strain.

The significantly lower expression of the developmental sigma factor BldN and its targets—three chaplins, NepA, and transferase—may be the result of OsdR expression, as OsdR is suggested to be a regulator of *bldN* expression [42]. The parallel downregulation of *ssgA*, a gene found to be essential for sporulation [60], could indicate a coordinated response. Both the decreased expression of these genes, and the potential presence of (increased) hypoxia in the CelA-producing strain could point to morphological changes, yet no observable morphological differences from the reference strain were recorded.

The link between the OsdR regulon and metabolic enzymes coupled to secondary metabolism (Table 2) was not previously reported. Isoenzymes of the PPP reactions are upregulated in the CelA-producing strain, which correlates well with the increased PPP fluxes. Whether this upregulation is actually required to support the flux increases is unclear. $^{13}\hbox {C}$-MFA on a deletion mutant for one or more of these isoenzymes could potentially address this.

Due to the increased flux through both the oxidative PPP and the TCA cycle, the NADPH yield per gram biomass is more than 50% higher in the CelA-producing strain. In *S. lividans*, only glucose-6-phosphate dehydrogenase is $\hbox {NADP}^+$-dependent and additional NADPH is generated in the TCA cycle, mainly via $\hbox {NADP}^+$-dependent isocitrate dehydrogenase activity and partly via 2-oxoglutarate dehydrogenase activity coupled to $\hbox {NADP}^+$-dependent ferroreductase. The redirection of carbon to the oxidative part of the PPP instead of glycolysis, which creates more NADPH, aligns with other studies on heterologous protein production in other micro-organisms, e.g., *Pichia pastoris* [61, 62], *Bacillus subtilis* [63], and *Aspergillus niger* [64, 65]. It was also demonstrated that overexpression of glucose-6-phosphate dehydrogenase (*zwf1*) and 6-gluconolactonase PPP (*sol3*) effectively increases the NADPH production in *Pichia pastoris*, which augments heterologous protein production [62]. Increased NADPH production can, however, only partly be explained by the need for extra NADPH for protein production, in this case including heterologous CelA protein as well as other secreted proteins. To keep cofactors balanced, i.e., to maintain redox balance, the excess NADPH in the CelA-producing strain needs to be regenerated to $\hbox {NADP}^+$. Since *S. lividans* TK24 lacks the cytoplasmic transhydrogenase UdhA (gene not present, [20]) which typically oxidizes NADPH into $\hbox {NADP}^+$, an alternate route must be present. Genes coding for the membrane-bound transhydrogenase PntAB are present in *S. lividans* TK24, but re-oxidation of NADPH through PntAB is thermodynamically not feasible [66]. Moreover, *pntA* and *pntB* expression levels in both the producing and reference strain are negligible. In many bacteria, PntAB supplies NADPH to balance requirements in anabolic reactions, which is not needed in our strains. A potential alternative for NADPH re-oxidation is NuoF (NADH-quinone oxidoreductase subunit F from NADH hydrogenase), which in *E. coli* was shown to alternatively function with NADPH and take over the function of UdhA upon its deletion [67]. However, no significant *nuoF* ($\hbox {SLIV}\_15430$) expression was observed in the exponential growth phase. Hence, no clear explanation can be given, and further studies are required to unravel how *S. lividans* handles NADPH overproduction. Overproduction of NADPH has been reported previously in overproducing *S. lividans* strains [68, 69]. The additional ATP created by the assumed re-oxidation of the surplus NADPH could partially be destined to supply ATP-dependent protein secretion via the Sec pathway SecA ATPase [1].

Overexpression of Gap3 clusters this gene with the OsdR regulon (Group III, Table 2), but does not correspond to a significant normalized net flux change from glyceraldehyde-3-phosphate to 2-phosphoglycerate (lumped reaction in the model). With Gap1 identified as the constitutive main glycolytic enzyme, and Gap2 the gluconeogenetic enzyme, the exact nature and function of Gap3 remains unknown. In other bacteria, $\hbox {NADP}^+$-dependent GADPH (GapN) has been reported [70], catalysing a one-step reaction from glyceraldehyde-3-phosphate to 3-phosphoglycerate. This would follow the general tendency to overproduce NADPH in case of heterologous protein production, yet no experimental evidence exists for its presence in *S. lividans*.

## Conclusions

In *S. lividans* TK24, heterologous production and secretion of CelA leads to a reduced growth rate, a distinct shift in the central carbon metabolism towards NADPH-production, and clear gene expression changes in subsets of genes correlating to CelA-production and the OsdR regulon. Transcriptomics data uncovered stress responses in the recombinant CelA-producing strain mostly related to secretory stress and DNA damage. The cause of this (perceived) DNA-damage and secretory stress is unclear and requires further study. The latter could indicate unknown bottlenecks in secretion, but identification of specific targets for strain improvement again requires additional research.

Isoenzymes linked to secondary metabolism are co-expressed with the OsdR regulon, and could be (partially) responsible for the measured flux increase through the PPP. Increased fluxes through both the PPP and the TCA lead to higher NADPH generation in a CelA-producing strain, which exceeds the amount needed for protein production. Redox balancing in the heterologous protein producing *S. lividans* fails and alternative routes are not yet fully understood. Further studies to the contribution of PPP isoenzymes and understanding transhydrogenase activity are required.

The findings presented here help build a foundation for strain improvement of the industrially important organism *S. lividans*.

## Additional files

**Additional file 1.** Description of the network model reconstruction for 13C-based metabolic flux analysis, and amino acid fragments used in 13C-MFA.

**Additional file 2.** Local clustering procedure.

**Additional file 3.** Network model in FTBL.

**Additional file 4.** Biomass and CelA composition used in 13C-based metabolic flux analysis.

**Additional file 5.** Gene expression data, differential expression data, local and hierarchical clustering data.

**Additional file 6.** Growth, metabolite, and protein secretion concentration profiles of 13C-MFA experiments.

**Additional file 7.** Mass isotopomer distribution (MID) measurements used for 13C-based flux estimation.

**Additional file 8.** Flux estimates, confidence intervals and flux maps for *S. lividans* with pIJ486 and *S. lividans* with pIJ486-vsi-celA with absolute flux values (mmol·gDW^−1^·h^−1^).

**Additional file 9.** Overview of enzymatic reactions and enzyme gene expression for the central carbon fluxes of the calculated fluxmaps.
