# Supplementary material for: Transcriptomic and fluxomic changes in Streptomyces lividans producing heterologous protein
Source: Microb Cell Fact. 2018 Dec 21;17:198. doi: 10.1186/s12934-018-1040-6 (PMC6302529; doi:10.1186/s12934-018-1040-6)
Supplement: Supplementary file 1 — Additional file 1. Description of the network model reconstruction for 13C-based metabolic flux analysis, and amino acid fragments used in 13C-MFA. [file 12934_2018_1040_MOESM1_ESM.pdf]

Network model reconstruction

The central carbon metabolism network model was based on the network model for *S. lividans* TK24 applied in [1]. Model improvements and changes were implemented: (*i*) revision of the biomass equation, (*ii*) incorporation of a cellulase A (CelA) and overall protein secretion efflux, (*iii*) addition of effluxes for organic acids, (*iv*) inclusion of CO_2 dilution flux according to [2], (*v*) removal of non-identifiable ammonia transfer reactions in the nitrogen metabolism, (*vi*) removal of the pyruvate carboxylase reaction flux, and (*vii*) adjustment to available measurements.

Metabolic precursors requirements were updated according to the most recently published macromolecular biomass composition of the closely related *Streptomyces coelicolor* [3]. The macromolecular composition was changed to include the plasmid DNA present in the cell. This resulted in a 4% increase in total DNA assuming a plasmid copy number of 50 [4]. Given the very small contribution of the *celA* gene in the total plasmid DNA, the biomass composition equation was kept the same for the CelA-producing strain. Metabolic precursors for the production of 1 gram of protein were re-evaluated—rectifying an incorrect interpretation of mole percentages in past studies—and reverted to the original amino acid composition as determined for *E. coli* [5]. C1-components were included as a metabolic precursor. Inclusion of the C1-components is, for example, a prerequisite for the correct biosynthesis of amino acids such as histidine and methionine. The final macromolecular composition is summarized in Additional file 4. The biomass equation was implemented as a measurable free flux (practically done by considering the alanine efflux as a free measured flux and defining dependent effluxes for the other biomass precursors scaled to the alanine efflux).

Effluxes for cellulase A (CelA), total secreted proteins, and a set of organic acids (i.e., acetic, α-ketoglutaric and pyruvic acid) were added. The efflux to pyruvic acid accounted for both lactic and pyruvic acid production. Effluxes of α-ketoglutaric acid, pyruvic acid, CelA, and total secrete protein were implemented as free fluxes with their experimentally determined yields and standard errors as measurements. Acetic acid was not included to allow a sink for carbon effluxes not accounted for in the measurements.

A dilution flux of CO_2 was added to account for the potential uptake of unlabeled CO_2 [2]. This dilution flux was implemented as a free flux and estimated during flux fitting.

The anaplerotic reaction from pyruvic acid to oxaloacetic acid was found to have a very limited expression, and was thus removed from the network.

Uptake fractions of the labelled glucose substrates were implemented as free fluxes, thereby accounting for imprecisions in mixture preparation, and the potential presence of 0-GLC remaining from the preculture medium. The labelled glucose mixture was thus set as a measurement expressing the feed mixture fractions. The uptake rate of glucose for both strains was fixed at a relative reference value of 100.

The model for *S. lividans* pIJ486 included 30 net fluxes (10 free and 20 dependent), and 13 (free) exchange fluxes (i.e., bidirectional flux through a reversible reaction without a net effect). The model for *S. lividans* pIJ486-CelA included one extra free net flux towards CelA. The network model and its implementation in FTBL-format can be found in Additional file 3.

Isotopic labelling data

Table 1: Amino acid fragments used in 13C-based MFA.

**Amino acids**	**Fragment**	**Mass**
Ala	C10H26NOSi2	232
Ala	C11H26NO2Si2	260
Asp	C14H32NO2Si2	302
Asp	C16H38NO3Si3	376
Asp	C17H40NO3Si3	390
Asp	C18H40NO4Si3	418
Glu	C16H36NO2Si2	330
Glu	C18H42NO3Si3	404
Glu	C19H42NO4Si3	432
Gly	C9H24NOSi2	218
Gly	C10H24NO2Si2	246
Ile	C11H26NSi	200
Ile	C13H32NOSi2	274
Leu	C13H32NOSi2	274
Phe	C14H24NSi	234
Phe	C14H32NO2Si2	302
Phe	C16H30NOSi2	308
Phe	C17H30NO2Si2	336
Ser	C14H34NOSi2	288
Ser	C16H40NO2Si3	362
Ser	C17H40NO3Si3	390
Thr	C17H42NO2Si3	376
Thr	C18H42NO3Si3	404
Tyr	C14H32NO2Si2	302
Val	C12H30NOSi2	260
Val	C13H30NO2Si2	288

References

[1] Bouvin, J., Cajot, S., D'Huys, P.J., Ampofo-Asiama, J., Anné, J., Van Impe, J., Geeraerd, A., Bernaerts, K.: Multi-objective experimental design for (13)C-based metabolic flux analysis. Math Biosci **268**, 22–30 (2015)

[2] Leighty, R.W., Antoniewicz, M.R.: Parallel labeling experiments with [U-13C]glucose validate *E. coli* metabolic network model for 13C metabolic flux analysis. Metab. Eng. **14**(5), 533–541 (2012)

[3] Coze, F., Gilard, F., Tcherkez, G., Virolle, M.J., Guyonvarch, A.: Carbon-flux distribution within *Streptomyces coelicolor* metabolism: a comparison between the actinorhodin-producing strain M145 and its non-producing derivative M1146. PLoS ONE **8**(12), 84151 (2013)

[4] Kieser, T., Bibb, M., Buttner, M.C., Hater, K., Hopwood, D.: Practical *Streptomyces* Genetics. The John Innes Foundation, Colney, Norwich NR4 7UH, England (2000)

[5] Ingraham, J.L., Maaløe, O., Neidhardt, F.C.: Growth of the Bacterial Cell, p. 435. Sinauer Associates, Sunderland, USA (1983)
