# Supplementary material for: Transcriptomic and fluxomic changes in Streptomyces lividans producing heterologous protein
Source: Microb Cell Fact. 2018 Dec 21;17:198. doi: 10.1186/s12934-018-1040-6 (PMC6302529; doi:10.1186/s12934-018-1040-6)
Supplement: Supplementary file 2 — Additional file 2. Local clustering procedure. [file 12934_2018_1040_MOESM2_ESM.pdf]

Local clustering algorithm documentation

Input

The local clustering algorithm uses a $n \times m$ gene expression matrix M containing TPM (transcripts per million) values, with n the number of measured genes and m the number of samples. Since clustering is dependent on genes' physical location on the genome, it is important that genes in the dataset are ordered by location and that the dataset is either gapless, or that the omitting of known genes is accounted for when calculating the physical distance between genes. The expression profile of one gene can be denoted by vector $\vec{O}_i = \{M_{ip} | 1 \leqslant p \leqslant m\}$.

Measuring gene similarity

For measuring the similarity between two genes, their TPM values are first normalized by z-scoring. The distance D_{ij} between two genes is then taken as the euclidean distance between their normalized expression vectors.

$$\vec{O}'_i = \frac{\vec{O}_i - \mu_{O_i}}{\sigma_{O_i}}$$

$$D_{ij} = \sqrt{\sum_{p=1}^{m} (O'_{ip} - O'_{jp})^2}$$

Distribution of distances

The total number of distances $D_{ij} (1 \leqslant i, j \leqslant n | i \neq j)$ in the dataset is:

$$d = \frac{n \times (n-1)}{2}$$

By determining and sorting the full set of distances we can obtain the cumulative distribution function (CDF) of distances, as illustrated in Figure 1). From this

CDF it is possible to find a p-value corresponding to a given distance and vice versa, which can be used as an indicator to determine if two genes are significantly co-expressed. If, for example, two genes are assumed to be co-expressed when their distance D_{ij} is in the lowest 1% of all distances, we can determine from the CDF illustrated in Figure 1 that D_{ij} needs to be 2.07 or less.

Note that it is more efficient to only store the distances corresponding to the low p-values. Furthermore, a random selection of genes from the dataset might suffice to generate a reliable CDF. This becomes important once the number of genes in the datasets grow very large.

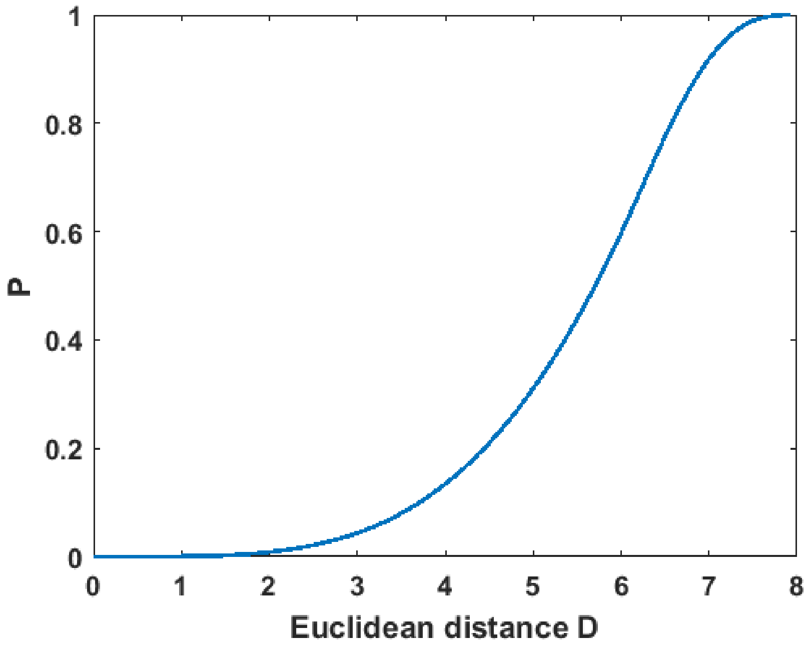

Figure 1: Cumulative distribution function of Euclidean distances between z-scored TPM (transcripts per million) values of a *Streptomyces lividans* gene expression dataset.

Location-based gene matching

With the obtained CDF, it is now possible to match all genes that are co-expressed with a certain p-value. However, in order to introduce dependence on genomic location in the matching process, the p-value required for matching

two genes is decreased as the physical distance on the genome increases. The distance between genes i and j in number of genes, $|i-j|$, and a base p-value p_0 is taken as the p-value required to match neighbouring genes, the specific cut-off p-value $p_{co,ij}$ for matching i and j is given by:

$$(1-p_{co,ij})^{|i-j|} = (1-p_0)$$

$$or$$

$$p_{co,ij} = 1 - e^{\frac{ln(1-p_0)}{|i-j|}} \tag{1}$$

This function is shown in Figure 2 for $p_0 = 0.01$. A toy example with 7 genes showing the location-based cut-off p-values for $p_0 = 0.01$ and resulting gene matches is given in Figure 3.

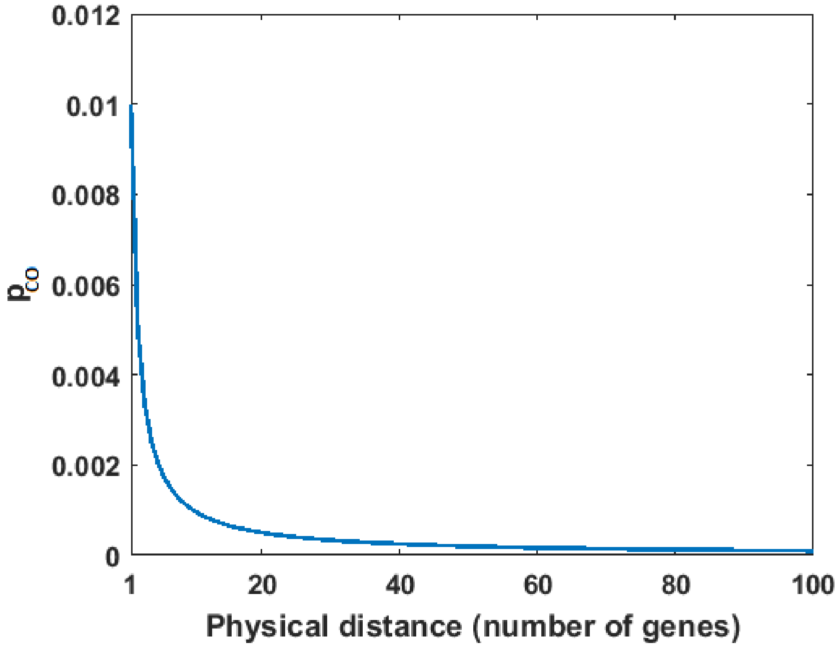

Figure 2: Dependence on physical location of cutoff p-value for matching two genes based on their expression profiles. As the physical distance between the two genes increases, the p-value drops and genes should have a more similar expression profiles to be matched. The base p-value p_0—the value required for matching two neighbouring genes—is 0.01 in this figure.

Forming and splitting clusters

The initial cluster forming procedure is straightforward: all genes that match directly or indirectly (through matches with other genes) are joined to the same cluster, which can be represented as a graph (Figure 3 B). This process can lead to large, heterogeneous clusters, especially for high values of p_0.

To ensure expression homogeneity within a cluster, all cluster genes are required to be co-expressed with a p-value lower than a given tolerance p_{tol}. If for genes i and j in the same cluster $p_{tol} \leqslant p_{ij}$, the cluster graph is split in two using a minimal cut algorithm, in which the weights of the edges for the matched genes (genes with a direct link) are given by:

$$W_{ij} = \left(\frac{D_{p=p_{co,ij}}}{D_{ij}} \right)^2$$

In which $D_{p=p_{co,ij}}$ is the cut-off distance for which genes i and j are matched, and D_{ij} is the actual distance between the genes. Note that in this equation, $D_{ij} \leqslant D_{p=p_{co,ij}}$, as otherwise the genes would not have been matched. For genes within the cluster that are not matched directly $W_{ij} = 0$.

After splitting a cluster, both resulting clusters are again examined for p-values higher than p_{tol}, and split further if necessary. Cluster splitting goes on until all genes within all resulting clusters meet the given p_{tol}.

If a cluster contains multiple pairs $X_{ij} = \{i, j | p_{tol} < p_{ij}\}$, a choice needs to be made as to which pair to split using the minimal cut algorithm, and the order of splitting different pairs will result in different clusters, and even different numbers of clusters. For large clusters, the number of gene-pairs that need to split can become very large, making it impossible to find a global optimal split (an NP-hard problem). The best results in terms total number of resulting clusters were obtained by first requiring a minimal cut between the pair with the smallest distance D_{ij}, as splitting this pair has the highest rate of splitting additional pairs of X, requiring a lower number of splits until all clusters meet the given criterion.

Results

Results are grouping of genes to clusters based on their expression profiles and their physical location on the genome. The maximal distance between two genes within one cluster is $D_{p=p_{tol}}$ (the distance corresponding to the given tolerance p-value). Note that not all genes are assigned to a cluster: genes that do not have nearby genes that are sufficiently co-expressed will remain isolated.

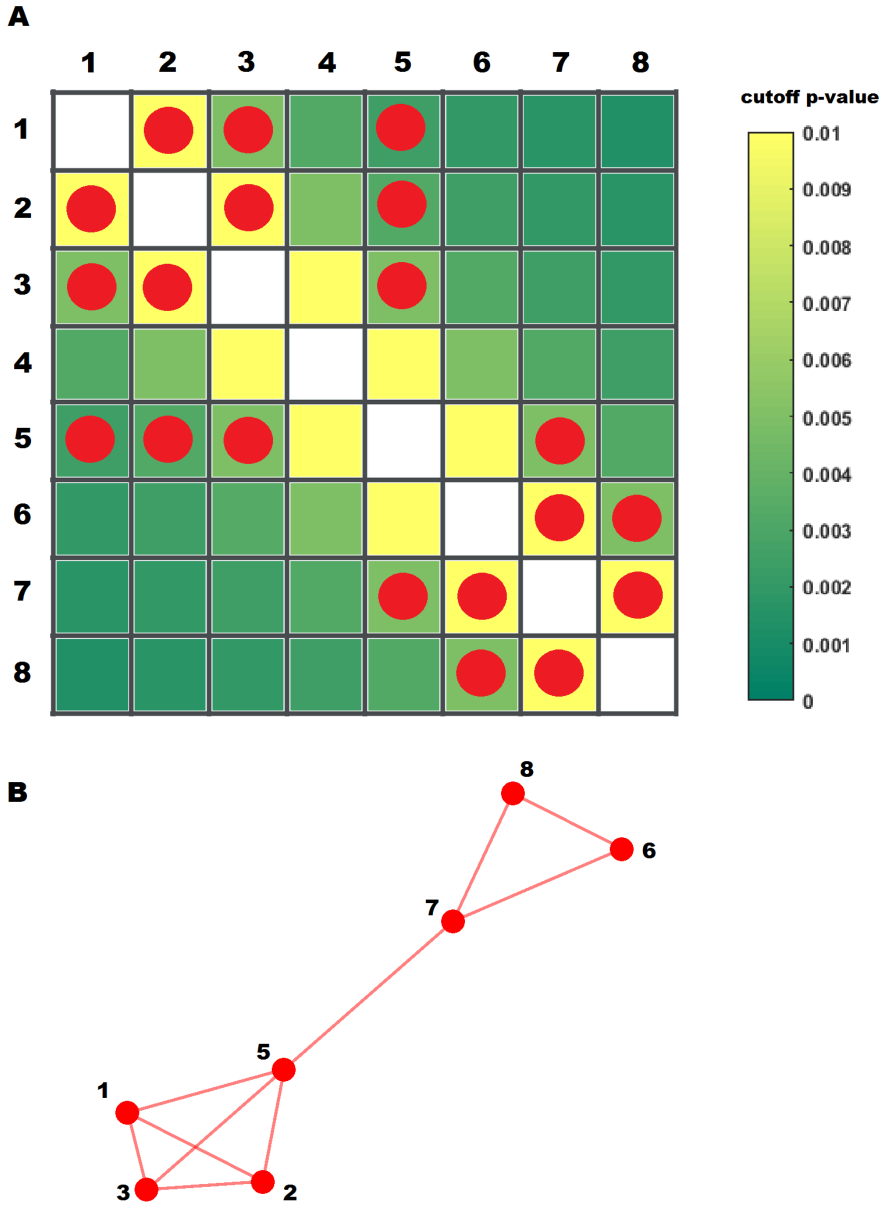

Figure 3: Toy example of gene matching mechanism for seven genes. (A) Requirements for matching genes increases (cut-off p-value decreases) as physical distance between genes increases. Exemplary gene matches are given by the red dots. Note that the matching pattern is always symmetrical. (B) The resulting cluster in the form of a graph in which the edges are the matches (direct links) between genes. The cluster contains all genes except gene 4.
