# Supplementary material for: Transcriptomic and fluxomic changes in Streptomyces lividans producing heterologous protein
Source: Microb Cell Fact. 2018 Dec 21;17:198. doi: 10.1186/s12934-018-1040-6 (PMC6302529; doi:10.1186/s12934-018-1040-6)

13C-MFA experimental data profiles

Streptomyces lividans TK24 *pIJ486* run 1

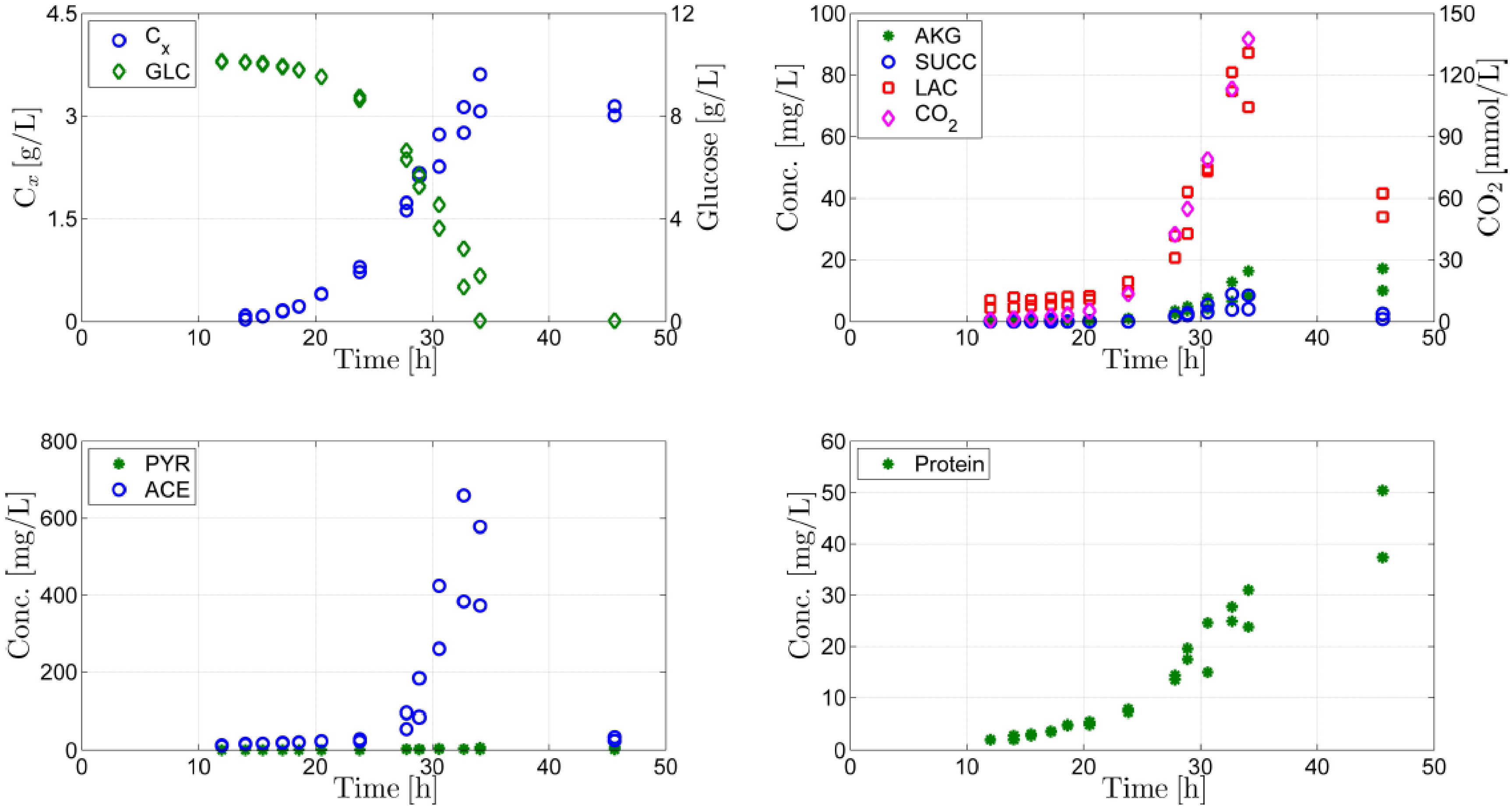

Streptomyces lividans TK24 *pIJ486* run 2

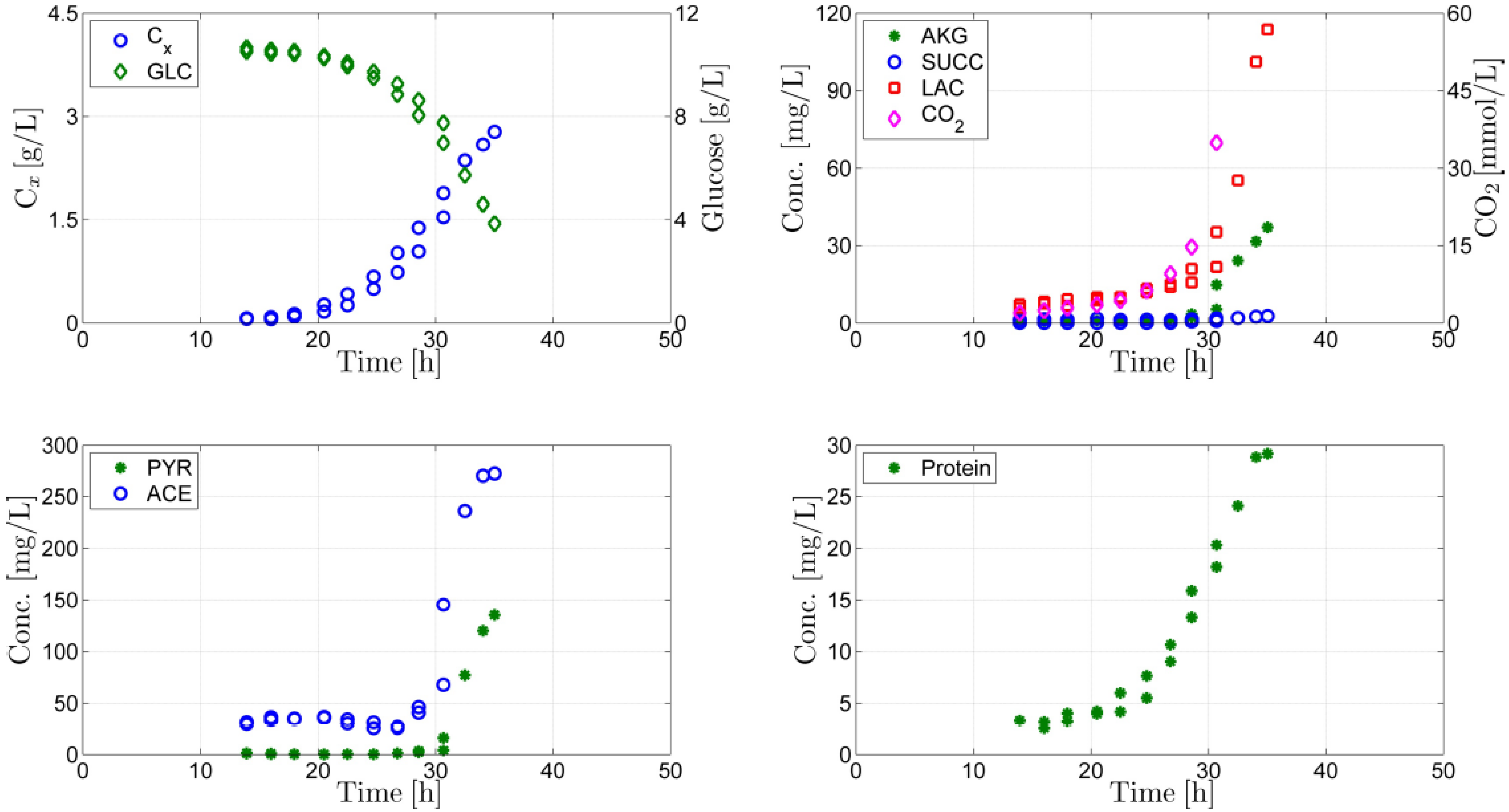

Streptomyces lividans TK24 *pIJ486-vsi-celA* run 1

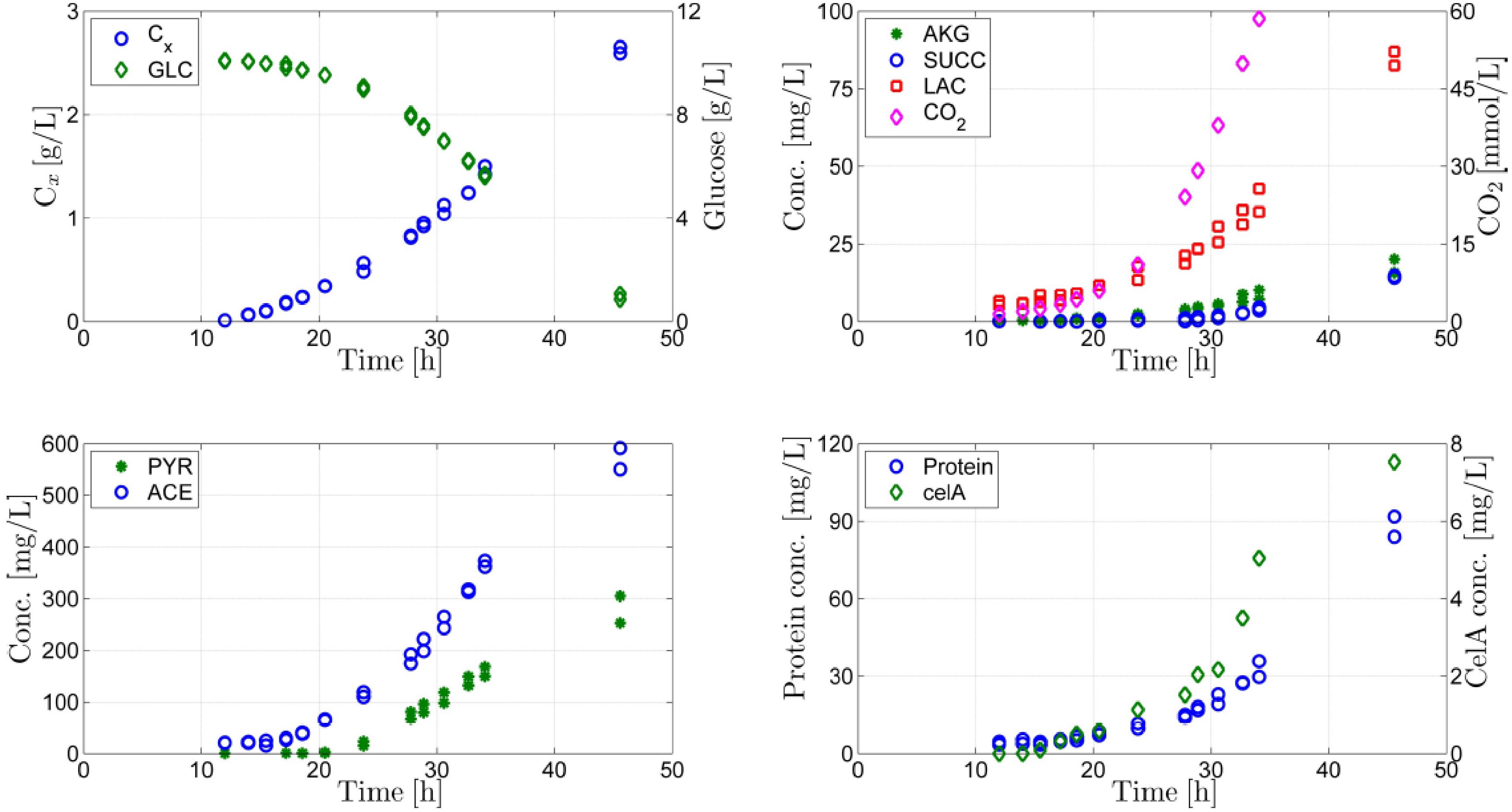

Streptomyces lividans TK24 *pIJ486-vsi-celA* run 2

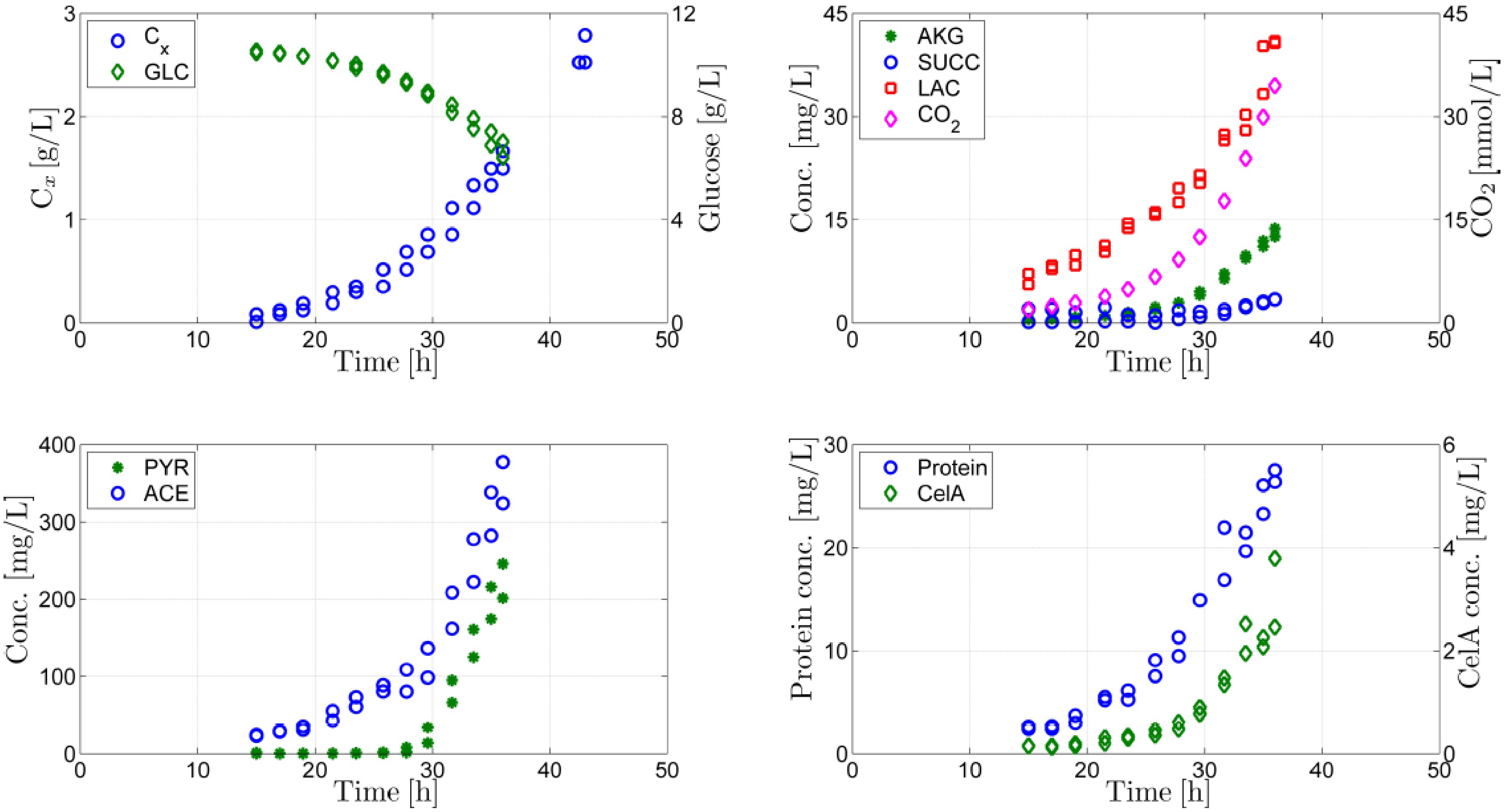

Supplement: Supplementary file 6 — Additional file 6. Growth, metabolite, and protein secretion concentration profiles of 13C-MFA experiments. [file 12934_2018_1040_MOESM6_ESM.pdf]
