# Supplementary material for: Transcriptomic and fluxomic changes in Streptomyces lividans producing heterologous protein
Source: Microb Cell Fact. 2018 Dec 21;17:198. doi: 10.1186/s12934-018-1040-6 (PMC6302529; doi:10.1186/s12934-018-1040-6)
Supplement: Supplementary file 8 — Additional file 8. Flux estimates, confidence intervals and flux maps for S. lividans with pIJ486 and S. lividans with pIJ486-vsi-celA with absolute flux values (mmol·gDW−1·h−1). [file 12934_2018_1040_MOESM8_ESM.pdf]

13C-based MFA: flux estimates

Table 1: Overview of estimated (free) net and exchange fluxes and associated confidence intervals for the CelA-producing strain and the empty-plasmid strain based on repeated 13C-experiments.

	***S. lividans* pIJ486**		***S. lividans* pIJ486-CelA**	
G6P $\rightarrow$ F6P	46.89	(42.28, 54.57)	23.78	(19.44, 32.64)
PEP $\rightarrow$ PYR	107.94	(103.24, 111.75)	129.04	(126.23, 132.40)
CO_2 dilution flux	8.99	(5.83, 12.81)	0.00	(0.00, 4.50)
ACE secretion	31.51	(22.51, 39.43)	34.31	(26.70, 43.89)
AKG secretion	0.11	(0.09, 0.13)	0.36	(0.29, 0.42)
PYR secretion	1.55	(1.09, 1.99)	9.84	(9.32, 10.45)
ALA $\rightarrow$ Biomass	3.16	(2.99, 3.32)	2.08	(1.98, 2.22)
ALA $\rightarrow$ CelA	-		0.02	(0.01, 0.03)
ALA $\rightarrow$ Protein	0.08	(0.06, 0.09)	0.09	(0.07, 0.11)
1-GLC uptake	44.01	(43.79, 44.20)	44.03	(43.84, 44.20)
U-GLC uptake	55.56	(55.44, 55.70)	55.63	(55.50, 55.77)
PEP + CO_2 $\leftrightarrow$ OAA	20.72	(15.01, 29.31)	38.43	(32.13, 44.01)
G6P $\leftrightarrow$ F6P	>1000	(353.13, ∞)	>1000	(528.62, ∞)
FBP $\leftrightarrow$ 2 GAP	>1000	(0.00, 131.68)	>1000	(0.00, 53.17)
GAP $\leftrightarrow$ PGA	>1000	(0.00, ∞)	>1000	(0.00, ∞)
PGA $\leftrightarrow$ PEP	>1000	(708.42, ∞)	>1000	(1645.78, ∞)
2 R5P $\leftrightarrow$ S7P + GAP	30.84	(4.41, 50.76)	48.13	(21.58, 90.53)
R5P + E4P $\leftrightarrow$ GAP + F6P	20.83	(13.99, 28.08)	4.29	(0.00, 11.51)
GAP + S7P $\leftrightarrow$ E4P + F6P	30.20	(22.49, 63.77)	8.87	(0.00, 44.77)
PYR $\leftrightarrow$ AcCoA + CO_2	9.42	(5.51, 12.46)	0.26	(0.00, 2.73)
AKG $\leftrightarrow$ SUCC + CO_2	0.00	(0.00, 6.07)	1.99	(0.00, 12.06)
SUCC $\leftrightarrow$ FUM	>1000	(0.00, ∞)	>1000	(0.00, ∞)
FUM $\leftrightarrow$ MAL	>1000	(57.35, ∞)	>1000	(55.36, ∞)
MAL $\leftrightarrow$ OAA	127.96	(119.84, ∞)	130.73	(124.55, ∞)

Table 2: Overview of all estimated net fluxes and their confidence interval in the central carbon metabolism of CelA-producing *S. lividans*and the empty plasmid reference strain. Both absolute flux values and normalised values are given. Estimated fluxes of biomass and protein are expressed in gram and milligram, respectively.

Fluxes		***CelA strain*** Normalised (mmol/100 mmol glucose)		***Reference strain*** Normalised (mmol/100 mmol glucose)		***CelA strain*** Absolute flux values (mmol/gDW·h)		***Reference strain*** Absolute flux values (mmol/gDW·h)	
Free fluxes									
**coIn**	CO_2 dilution	0.00	(0:4.50)	8.99	(5.83:12.81)	0.00	(0:0.09)	0.22	(0.14:0.31)
**Eff_Ac**	Secretion Ac	34.31	(26.7:43.89)	31.51	(22.51:39.43)	0.68	(0.53:0.87)	0.76	(0.54:0.95)
**Eff_AKG**	Secretion AKG	0.36	(0.29:0.42)	0.11	(0.09:0.13)	0.01	(0.01:0.01)	0	(0:0)
**Eff_PYR**	Secretion PYR	9.84	(9.32:10.45)	1.55	(1.09:1.99)	0.19	(0.18:0.21)	0.04	(0.03:0.05)
**emp1**	G6P → F6P	23.78	(19.44:32.64)	46.89	(42.28:54.57)	0.47	(0.38:0.65)	1.13	(1.02:1.32)
**emp6**	PEP → PYR	129.04	(126.23:132.4)	107.94	(103.24:111.75)	2.55	(2.5:2.62)	2.61	(2.49:2.7)
**Biomass efflux**	[gram]	5.72	(5.45:6.11)	8.67	(8.22:9.13)	0.11	(0.11:0.12)	0.21	(0.2:0.22)
**CelA secretion**	[milligram]	18.53	(9.27:27.8)			0.37	(0.18:0.55)		
**Protein secretion**	[milligram]	104.33	(81.15:127.51)	86.10	(71.58:99.74)	2.07	(1.61:2.52)	2.08	(1.73:2.41)
Dependent fluxes									
**ana1**	MAL → PYR + CO_2	0.03	(0:2.48)	6.58	(4.1:9.84)	0.00	(0:0.05)	0.16	(0.1:0.24)
**ana3**	PEP +CO_2 → OAA	15.25	(14.57:17.69)	28.82	(26.17:32.05)	0.30	(0.29:0.35)	0.7	(0.63:0.77)
**coExch**	CO_2 dilution	0.00	(0:5.67)	8.99	(5.83:12.81)	0.00	(0:0.11)	0.22	(0.14:0.31)
**coOut**	CO_2 production	283.04	(261.48:296.53)	206.94	(194.83:220.71)	5.60	(5.18:5.87)	5	(4.7:5.33)
**emp2**	F6P → FBP	*67.97*	*(66.46:71.29)*	72.31	(70.94:74.66)	*1.35*	(1.32:1.41)	1.75	(1.71:1.8)
**emp3**	FBP → 2 GAP	67.97	(66.46:71.29)	72.31	(70.94:74.66)	1.35	(1.32:1.41)	1.75	(1.71:1.8)
**emp4**	GAP → PGA	155.23	(153.31:158.83)	153.09	(151.3:155.89)	3.07	(3.04:3.14)	3.7	(3.65:3.76)
**emp5**	PGA → PEP	148.14	(145.88:151.91)	142.45	(140.36:145.49)	2.93	(2.89:3.01)	3.44	(3.39:3.51)
**ppp1**	G6P → R5P	75.27	(65.95:80.13)	51.66	(44:56.35)	1.49	(1.31:1.59)	1.25	(1.06:1.36)
**ppp4**	2 R5P → S7P + GAP	23.94	(20.88:25.58)	15.47	(12.93:17.12)	0.47	(0.41:0.51)	0.37	(0.31:0.41)
**ppp5**	R5P + E4P → F6P + GAP	22.22	(19.23:23.89)	12.93	(10.44:14.59)	0.44	(0.38:0.47)	0.31	(0.25:0.35)
**ppp6**	S7P + GAP → E4P + F6P	23.94	(20.88:25.58)	15.47	(12.93:17.12)	0.47	(0.41:0.51)	0.37	(0.31:0.41)
**tca1**	PYR → AcCoA + CO_2	103.98	(99.54:108.59)	90.30	(85.7:94.98)	2.06	(1.97:2.15)	2.18	(2.07:2.29)
**tca2**	AcCoA + OAA → CitiCit	58.03	(50.13:63.56)	41.22	(37.24:47.51)	1.15	(0.99:1.26)	1	(0.9:1.15)
**tca3**	CitiCit → AKG + CO_2	58.03	(50.13:63.56)	41.22	(37.24:47.51)	1.15	(0.99:1.26)	1	(0.9:1.15)
**tca4**	AKG → SUCC +CO_2	52.51	(44.7:57.8)	33.43	(29.33:39.64)	1.04	(0.89:1.14)	0.81	(0.71:0.96)
**tca6**	SUCC → FUM	52.51	(44.7:57.8)	33.43	(29.33:39.64)	1.04	(0.89:1.14)	0.81	(0.71:0.96)
**tca7a**	FUM → MAL	26.25	(22.35:28.9)	16.72	(14.67:19.82)	0.52	(0.44:0.57)	0.4	(0.35:0.48)
**tca7b**	FUM → MAL	26.25	(22.35:28.9)	16.72	(14.67:19.82)	0.52	(0.44:0.57)	0.4	(0.35:0.48)
**tca8**	MAL → OAA	52.47	(44.07:57.76)	26.86	(21.86:31.61)	1.04	(0.87:1.14)	0.65	(0.53:0.76)
